# Paneth cell dysfunction in radiation injury and radio-mitigation by human α-defensin 5

**DOI:** 10.3389/fimmu.2023.1174140

**Published:** 2023-08-10

**Authors:** Pradeep K. Shukla, Roshan G. Rao, Avtar S. Meena, Francesco Giorgianni, Sue Chin Lee, Preeti Raju, Nitesh Shashikanth, Chandra Shekhar, Sarka Beranova, Louisa Balazs, Gabor Tigyi, Ankush Gosain, RadhaKrishna Rao

**Affiliations:** ^1^ College of Medicine, University of Tennessee Health Science Center, Memphis, TN, United States; ^2^ College of Pharmacy, University of Tennessee Health Science Center, Memphis, TN, United States

**Keywords:** GI-ARS, irradiation, defensins, tight junction, intestine, barrier function, microbiome

## Abstract

**Introduction:**

The mechanism underlying radiation-induced gut microbiota dysbiosis is undefined. This study examined the effect of radiation on the intestinal Paneth cell α-defensin expression and its impact on microbiota composition and mucosal tissue injury and evaluated the radio-mitigative effect of human α-defensin 5 (HD5).

**Methods:**

Adult mice were subjected to total body irradiation, and Paneth cell α-defensin expression was evaluated by measuring α-defensin mRNA by RT-PCR and α-defensin peptide levels by mass spectrometry. Vascular-to-luminal flux of FITC-inulin was measured to evaluate intestinal mucosal permeability and endotoxemia by measuring plasma lipopolysaccharide. HD5 was administered in a liquid diet 24 hours before or after irradiation. Gut microbiota was analyzed by 16S rRNA sequencing. Intestinal epithelial junctions were analyzed by immunofluorescence confocal microscopy and mucosal inflammatory response by cytokine expression. Systemic inflammation was evaluated by measuring plasma cytokine levels.

**Results:**

Ionizing radiation reduced the Paneth cell α-defensin expression and depleted α-defensin peptides in the intestinal lumen. α-Defensin down-regulation was associated with the time-dependent alteration of gut microbiota composition, increased gut permeability, and endotoxemia. Administration of human α-defensin 5 (HD5) in the diet 24 hours before irradiation (prophylactic) significantly blocked radiation-induced gut microbiota dysbiosis, disruption of intestinal epithelial tight junction and adherens junction, mucosal barrier dysfunction, and mucosal inflammatory response. HD5, administered 24 hours after irradiation (treatment), reversed radiation-induced microbiota dysbiosis, tight junction and adherens junction disruption, and barrier dysfunction. Furthermore, HD5 treatment also prevents and reverses radiation-induced endotoxemia and systemic inflammation.

**Conclusion:**

These data demonstrate that radiation induces Paneth cell dysfunction in the intestine, and HD5 feeding prevents and mitigates radiation-induced intestinal mucosal injury, endotoxemia, and systemic inflammation.

## 1 Introduction

Exposure to high-dose ionizing radiation results in a complex multi-organ injury, a condition referred to as Acute Radiation Syndrome (ARS). The pathogenesis of ARS is multisystemic and associated with high morbidity and mortality. ARS is characterized by an immediate effect of radiation on the hematopoietic system (H-ARS) and gastrointestinal tract (GI-ARS). GI-ARS is characterized by nausea, diarrhea, and vomiting, but no FDA-approved drugs are available to treat GI-ARS. Although the precise mechanism involved in GI-ARS pathogenesis is unclear, acute irradiation is known to suppress the mucosal immune system (1–3), alter gut microbiota composition (increased pathobionts and reduced beneficial bacteria) (4–8), and cause mucosal barrier dysfunction by disrupting the epithelial junctions (9). Microbiota dysbiosis and epithelial barrier dysfunction result in bacterial lipopolysaccharides (LPS) translocation into the circulation, causing endotoxemia. Endotoxemia leads to systemic inflammation and multi-organ injury (10–14). Therefore, GI-ARS has a global impact on the irradiated body.

Human studies concerning radiotherapy of cancer patients demonstrate a correlation between radiation enteritis and dysbiosis of gut microbiota (15–17). Such studies have shed light on the potential microbiome changes in accidental human exposure to high-dose radiation and have been confirmed in numerous studies using animal models of GI-ARS (8, 18–20). Altered microbiota plays a significant role in intestinal response to radiation (7, 8, 21, 22). Radiation-induced dysbiosis is characterized by low microbiota diversity with decreased abundance of beneficial bacteria, and increased pathobionts (23). However, the mechanism by which radiation alters microbiota composition is poorly defined. A potential mechanism may involve the suppression of intestinal mucosal immune function.

Ionizing radiation impacts the immune system (24), and immune dysfunction persists in survivors for decades after radiation exposure (25, 26). The GI mucosal immune system is highly susceptible to radiation damage (27); however, the precise mechanism of radiation effects on the innate mucosal immune function remains to be investigated. The innate mucosal immune system consists of epithelial cells (enterocytes, goblet, and Paneth cells) (28) and sub-epithelial macrophages and neutrophils (29–31). Paneth cells are the unique secretory cells at the base of the intestinal crypts of Lieberkühn (32–34). These cells form an integral part of the innate immune system (28, 31, 35, 36) by producing and secreting antimicrobial proteins, such as α-defensins (28, 37–39). α-Defensins are a family of cationic peptides (40) that form an integral part of the innate immune system (31, 37, 41). Whereas mouse intestinal Paneth cells produce six isoforms of α-defensins (also known as cryptdins) (42–44), human Paneth cells secrete two α-defensins known as human defensin 5 (HD5) and human defensin 6 (HD6) (44, 45). HD5 is the primary antimicrobial peptide released into the human intestinal lumen, which plays a role in maintaining a balanced microbiota composition (35, 36, 46–48). Depletion of α-defensins is associated with gut microbiota dysbiosis and endotoxemia (49, 50). Total body irradiation at a sublethal dose decreases intestinal mucosal macrophages, neutrophils, and lymphocytes (27). However, the effect of irradiation radiation on Paneth cell function is unknown.

Another critical factor required for developing endotoxemia is the disruption of epithelial tight junctions (TJ), leading to the loss of mucosal barrier function and translocation of LPS (51–56). Epithelial tight junctions comprise transmembrane proteins such as occludin, junctional adhesion molecule, claudins, and tricellulin, which bind to adapter proteins such as ZO-1 and plaque proteins such as cingulin (57). The extracellular domains of occludin and claudins interact with similar domains in the adjacent cells to occlude plasma membranes of adjacent cells, thus forming a physical barrier to prevent diffusion of macromolecules across the epithelium. Adherens junction (AJ), another epithelial junction located beneath the TJ, is formed by multiple proteins such as E-cadherin, catenins, and actin-binding proteins. AJ does not form a physical barrier for molecular movement across the epithelium, but it indirectly regulates the integrity of TJ. Recent studies have shown that total body irradiation in mice induces rapid disruption of TJ and causes mucosal barrier dysfunction (9, 58).

This study was designed to determine the effect of high-dose ionizing radiation on the intestinal Paneth cell α-defensins and its impact on the gut microbiota composition, gut barrier function, endotoxemia, and systemic inflammation.

## 2 Materials and methods

### 2.1 Materials

Maltose dextrin was purchased from Bioserv (Flemington, NJ; Cat# 3585), and Lieber DeCarli liquid diet (Dyets no. 710260) was procured from Dyets Inc. (Bethlehem, PA). Hoechst 33342 dye was purchased from Thermo Fisher Scientific (Waltham, MA; #62249). 33-1500 (clone oc-3f10) and anti-occludin (#33-1500) antibodies were obtained from Invitrogen (Carlsbad, CA). Anti-E-cadherin antibody (#14-3249-82) was purchased from Thermo Fisher Scientific, and anti-β-catenin antibody (#PA5-77934) was obtained from Invitrogen. AlexaFlour-488-conjugated anti-mouse IgG (#AP127P) and Cy3-conjugated anti-rabbit IgG (#12-348) were purchased from Millipore Sigma (Burlington, MA). Guanidinium chloride was purchased from Millipore Sigma, and reduced (#70-18-8) and oxidized (#27025-41-8) glutathione was procured from Sigma Aldrich. All other chemicals were purchased from Sigma-Aldrich (St Louis, MO) or Thermo Fisher Scientific (Tustin, CA).

### 2.2 HD5 preparation

HD5 was custom synthesized by Biomatik Inc. (Wilmington, DE), purified by HPLC, and authenticated by LC-MS/MS analysis. The peptide was dissolved in 8 M guanidium chloride (GuHCl) containing a mixture of reduced (3 mM) and oxidized (0.3 mM) glutathione (GSH), followed by dilution with 0.25 M sodium bicarbonate (NaHCO3) to adjust pH to 8.3 and incubated overnight for folding at room temperature. The working concentrations of HD5 and GuHCL were 0.5 mg/ml and 2 M, respectively. The final vehicle consists of GuHCl (6.4 M), reduced GSH (3 mM), oxidized GSH (0.3 mM), and NaHCO3 (0.05 M).

### 2.3 Animals and experimental design

All animal studies were performed under protocols approved by the University of Tennessee Health Science Center Institutional Animal Care and Use Committee. Adult mice (10-12 weeks old, C57BL6 mice purchased from Envigo, Indianapolis, IN) were subjected to irradiation or sham treatment with or without HD5.

*Study 1*: Mice were randomized into six groups, and five of them were subjected to total body irradiation (IR; 9.5 Gy at a dose rate of ~76 cGy/min), and one group was treated similarly without irradiation (Sham). At 4 h, 24 h, 48, 96 h, and 168 h, one group of mice was analyzed for intestinal Paneth cell α-defensin expression, intestinal mucosal permeability *in vivo*, microbiota composition, and endotoxemia. In addition, the sham group of mice was analyzed 24 h after treatment.

*Study 2*: Mice were randomized into four groups and fed a liquid diet with vehicle (as described above) or HD5 (6.6 mg/L diets; 1.8 μM; equivalent to 5 mg/kg BW/day based on an average diet intake of 0.7 mL/g BW/day) for 24 hours. Our previous *in vitro* study showed that HD5 produced the optimal antibacterial activity at a dose of 2 μM (59). Each vehicle and HD5-treated animal were subjected to sham treatment (Veh-Sham and HD5-Sham) or irradiation (Veh-IR and HD5-IR). At 24 hours after irradiation, gut microbiota composition, TJ and AJ integrity, mucosal barrier function, inflammatory response, endotoxemia, and systemic inflammation were analyzed.

*Study 3*: This study was similar to Study-2, except that Vehicle and HD5 were administered in the liquid diet 24 hours after irradiation. Animals were subjected to analysis 24 hours after HD5 treatment.

### 2.4 Irradiation

Mice were subjected to total body 9.5 Gy γ-irradiation from a ^137^Cs source (using a J.L. Shepherd & Assoc. Mark I, Model 25, San Fernando, CA, USA) at ~76 cGy/min. Radiation field mapping and calibration by ion chamber dosimetry were done by the manufacturer. Also, a certified health physicist conducted routine validation and quality control measurements of exposure rates and exposure rate mapping in the chamber at positions of interest using a calibrated RadCal 0.6 cc therapy grade ion chamber/electrometer system. High-dose thermo-luminescent dosimeters were used in most irradiations to validate the actual dose delivered to the mice (calibrated by MD Anderson Cancer Center Radiation Dosimetry Services). At the end of the experiment, gut permeability was measured as described below.

### 2.5 Gut permeability *in vivo*

Intestinal permeability to FITC-inulin (6 kDa) was measured *in vivo* as described before (60) to evaluate the mucosal barrier dysfunction. At the end of the experiment, mice were injected with FITC-inulin (6 kDa MW; 50 mg/ml solution; 2 µl/g body weight) *via* the tail vein. Blood samples were collected by cardiac puncture under isoflurane anesthesia one hour after the FITC-inulin injection. Plasma samples were prepared using a heparin sulfate anticoagulant. Colon and ileum luminal contents were flushed with 0.9% saline. A fluorescence plate reader mention instrument, wavelengths was used to measure fluorescence in plasma and luminal flushing. Fluorescence values were calculated as the percent of the amount injected by normalizing them to plasma fluorescence values.

There are multiple methods to evaluate gut permeability and intestinal mucosal barrier function. However, every method has its advantages and disadvantages. Since permeability through disrupted TJ has no directionality, and the transepithelial transport of macromolecules by passive diffusion, luminal-to-vascular and vascular-to-luminal permeability of FITC-inulin are expected to produce similar results. While the vascular-to-luminal flux differs from the luminal-to-vascular flux of LPS, we have found this method consistent with the TJ integrity analysis by immunofluorescence confocal microscopy. The oral gavage method does not distinguish the segment of the intestine where not needed barrier function is altered, and the absorption measured by this method is altered by changes in gastrointestinal motility.

### 2.6 Microbiota composition

The microbiome composition of colonic flushing was analyzed by 16S rRNA sequencing and metagenomic analyses, as described recently (60–62).

#### 2.6.1 DNA extraction and Illumina MiSeq sequencing

Samples were resuspended in 500 μL of TNES buffer containing 200 units of lyticase and 100 μL of 0.1/0.5 (50/50 Vol.) zirconia beads. Incubation was performed for 20 min at 37°C. Following mechanical disruption using ultra-high-speed bead beating, 20 μg of proteinase K was added to all samples, and they were incubated overnight at 55°C with agitation. Total DNA was extracted using phenol:chloroform:isoamyl alcohol mixture (25:24:1), and total DNA concentration per mg stool was determined by qRT-PCR. Purified DNA samples were sent to the Microbiome Resource, the University of Alabama (Birmingham, AL), for amplicon sequencing using the NextGen Illumina MiSeq platform. Blank samples passed through the entire collection, extraction, and amplification process and remained free of DNA amplification.

#### 2.6.2 Bioinformatics

Sequencing data were processed and analyzed using QIIME (Quantitative Insights into Microbial Ecology) 1.9.1 and Calypso 8.84 (63). The Shannon index was applied to quantify α-diversity (64, 65). Bray-Curtis analysis was used to quantify β-diversity, and the differences were compared using PERMANOVA with 999 permutations. We adjusted ANOVA using the Bonferroni correction and FDR for multiple comparisons to quantify the differences in the relative abundance of taxa between groups (66). The significance and high-dimensional biomarker identification were performed by linear discriminant analysis of effect size (LEfSe) (67).

### 2.7 Immunofluorescence microscopy

Immunofluorescence staining of TJ and AJ proteins was performed as described before (68). Cryo-sections of the colon (10 µm thickness) were fixed in acetone:methanol mixture (1:1) at -20°C for two minutes and rehydrated in 14 mM phosphate-buffered saline (PBS). Next, sections were permeabilized with 0.5% Triton X-100 in PBS for 15 minutes and blocked in 4% non-fat milk in TBST (20 mM Tris, pH 7.2, and 150 mM NaCl). Tissues were first incubated with primary antibodies (mouse monoclonal anti-occludin and rabbit polyclonal anti-ZO-1 antibodies or mouse monoclonal E-cadherin and rabbit polyclonal anti-β-catenin antibodies) for one hour. Unbound primary antibodies were washed off, and the tissues were incubated with the secondary antibodies (AlexaFluor 488-conjugated anti-mouse IgG, #A-11001, and Cy3-conjugated anti-rabbit IgG, #A-10520, antibodies from ThermoFisher Scientific, Tustin, CA) containing Hoechst 33342 dye for an additional hour. Images from x-y (1 μm) sections were captured using LSM Pascal or Zen software (White Plains, NY, USA). Images from optical sections were stacked using ImageJ software (NIH, Bethesda, MD, USA) and processed with Adobe Photoshop (Adobe Systems, San Jose, CA, USA). Optimal conditions of laser strength, gain, and contrast for the intestinal sections were first determined in the Sham group of mice; all other images within the experiment were captured using the same conditions. Images were processed in Image J and Adobe Photoshop software using identical conditions for all groups of images so that quantitative comparisons were not compromised.

### 2.8 α-Defensin peptide analysis

Peptides in colonic flushing were extracted in 30% acetic acid at 4°C for 30 min and centrifugation at 5000 x g for 5 min. The supernatant was lyophilized and extracted in HPLC-grade water. α-Defensin peptides were measured by mass spectrometry and proteomics by leveraging a new cutting-edge, high-resolution ion mobility tandem mass spectrometer (Synapt G2-*Si*; Waters Corporation) (69, 70). Peptides in the colonic extracts were purified in Oasis HLB solid-phase extraction cartridges (Waters) and digested with sequencing-grade trypsin (Promega) using established protocols (70). Samples were analyzed on an Acquity UPLC M-Class nano-LC system (Waters) interfaced with a Quadrupole Time-of-flight (QTof) tandem mass spectrometer with ion mobility separation (IMS) (Synapt G2-*Si*). The data were analyzed with Progenesis QI for Proteomics software.

### 2.9 RNA extraction and RT-qPCR

RNA was isolated from the ileum and colon using the TRIzol kit (Invitrogen, Carlsbad, CA, USA) and quantified using a NanoDrop photometer as described before (59). From the total RNA (1.5 μg), cDNA was generated using the ThermoScript RT-PCR kit for first-strand synthesis (Invitrogen). Quantitative PCR (qPCR) reactions were performed using cDNA mix (cDNA corresponding to 35 ng RNA) with 300 nmole primers in a final volume of 25 μl of 2× concentrated RT2 Real-Time SYBR Green/ROX master mix (Qiagen, Germantown, MD, USA) in an Applied Biosystems QuantStudio 6 Flex Real-Time PCR instrument (Norwalk, CT, USA). The cycle parameters were 50°C for 2 min, one denaturation step at 95°C for 10 min, and 40 cycles of denaturation at 95°C for 10 s, followed by annealing and elongation at 60°C. Gene expression of each transcript was normalized to the GAPDH gene transcripts using the ΔΔCt method. Primer sequences (α-defensins, cytokines, and microbiota genes) are provided in the **Supplemental Information** section (**Supplemental Information**, **Table-S1**, **S2**).

### 2.10 Plasma endotoxin assay

The Pierce LAL Chromogenic Endotoxin Quantitation Kit (Thermo Scientific, Cat# 88282) according to the manufacturer’s instructions to measure plasma endotoxin concentrations.

### 2.11 Plasma cytokine assay

Plasma cytokine and chemokine levels were measured using a Duoset ELISA kit (R&D system, Minneapolis, MN) according to vendor’s instructions. Briefly, plasma samples (50 μl) were first incubated in the capture antibody-coated microplates overnight and then incubated for 2 hours with the detection antibody. Following detection with the detection antibody, plates were incubated for 20 min with horseradish peroxidase-conjugated streptavidin and then with substrate solution for 20 min. The reaction was terminated by stop-solution, and absorbance was measured at 450 nm with a wavelength correction at 570 nm.

### 2.12 Histopathology

The distal colon was fixed in 10% buffered formalin (Sigma Aldrich) for 24 h, and 6 µm thick sections were collected using microtome onto glass slides and stained with hematoxylin and eosin (H & E). Following dehydration by graded ethanol washes (50%, 70%, 95%, 100%) and xylene washes, sections were mounted with a permanent mounting medium (Vector Laboratories). The bright-field images were captured at 10X magnification using a Nikon Eclipse Ti microscope (Melville, NY). A board-certified anatomic pathologist performed the blinded histological evaluation. The histological changes were graded as 0 for no abnormality, 1 for mild, 2 for moderate, and 3 for severe.

### 2.13 Statistical analysis

All data are expressed as mean ± SEM. ANOVA was applied for the analysis of differences among multiple groups. Statistical significance was assessed by 1-way ANOVA and Tukey’s *post hoc* test. This statement replaces the previous statement. Statistical analyses were performed using GraphPad Prism 9 software (San Diego, CA, USA), which uses the Omnibus K2 test for normality. The comparison of groups with uneven samples was confirmed with Welch’s t-test. Statistical significance was established at 95% confidence (p values <0.05).

## 3 Results

### 3.1 Ionizing radiation down-regulates intestinal α-defensins and alters gut microbiota leading to endotoxemia

**Figure 1:**
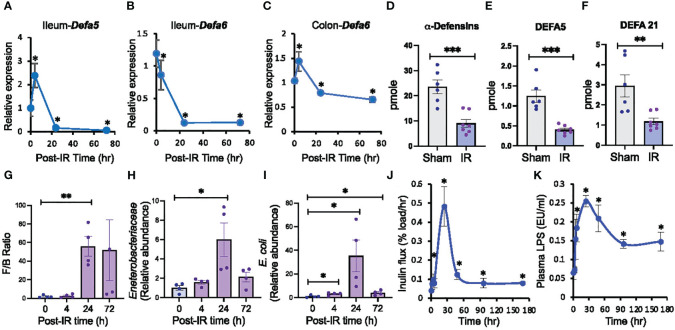
Ionizing radiation down-regulates Paneth cell α-defensins, alters gut microbiota, increases gut permeability, and leads to endotoxemia. Adult mice were exposed to ionizing radiation (IR) or sham-treated (0 hour). Intestinal α-defensin expression **(A-F)**, microbiota composition **(G-I)**, mucosal permeability **(J)**, and plasma LPS **(K)** were analyzed at varying times after irradiation (post-IR). **(A-C)** RNA preparations from the ileum and colon were analyzed for *Defa5* **(A, B)** and *Defa6* **(C)** mRNA by RT-qPCR. Zero-hour values represent the sham-treated group. Values are mean ± sem (n = 6); *p<0.05 for significant difference from “0 h” value. **(D-F)** Colonic luminal flushing from Sham-treated and irradiated mice at 24 hours after irradiation were analyzed for overall α-defensins **(D)**, DEFA5 **(E)**, and DEFA21 **(F)** by mass spectrometry. Values are mean ± sem (n = 6); **p<0.01, ***p<0.001 for significant difference from corresponding “Sham” values. **(G-I)** At varying times after irradiation, DNA preparations from colonic luminal flushing were analyzed for selected microbiota taxa by RT-qPCR. Results of *Firmicutes*/*Bacteroidetes* or F/B ratio **(G)**, *Enterobacteriaceae* **(H)**, and *E*. *coli* **(I)** by mass spectrometry. Zero-hour values represent the sham-treated group. Values are mean ± sem (n = 4); *p<0.05, **p<0.01 for significant difference from corresponding “0 h” values. **(J)** At varying times after irradiation, colonic mucosal permeability *in vivo* was evaluated by measuring the vascular-to-luminal flux of FITC-inulin. Zero-hour values represent the sham-treated group. Values are mean ± sem (n = 6); *p<0.05 for significant difference from corresponding “0 h” value. **(K)** Plasma LPS levels were measured at varying times after irradiation. Zero-hour values represent the sham-treated group. Values are mean ± sem (n = 6); *p<0.05 for significant difference from corresponding “0 h” value.

Radiation-induced dysbiosis of gut microbiota is a well-established fact. However, the precise mechanism involved in this effect is unclear. To determine the potential role of compromised innate mucosal immunity in radiation-induced gut microbiota dysbiosis, we evaluated the effect of 9.5 Gy γ-irradiation from a ^137^Cs source on the expression of Paneth cell α-defensins. *Defa5* (**Figure 1A**) and *Defa6* (**Figure 1B**) mRNA levels in the ileum were significantly decreased by radiation and remained low at least 72 hours post-irradiation; *Defa5* mRNA was transiently elevated before its decline. Paneth cells are scarce in the colon, and α-defensin expression is confined to the most proximal part of the colon in rats (71). In the irradiated mice colons, *Defa5* mRNA was not detectable, but *Defa6* mRNA was reduced (**Figure 1C**). α-Defensins are secreted by the small intestine, which flows into the colonic lumen and controls the microbiota composition in the colon. We developed a novel method using mass spectrometry and proteomics to analyze α-defensin quantity in the colonic luminal contents. Data show that radiation depletes α-defensins in the colon (**Figure 1D**), including DEFA5 (**Figure 1E**) and DEFA21 (**Figure 1F**). To determine whether the α-defensin down-regulation is associated with the alteration of microbiota composition, we assessed the microbiota composition by RT-qPCR. The *Firmicutes*/*Bacteroidetes* ratio was increased 24 hours post-irradiation (**Figure 1G**). The relative abundance of *Enterobacteriaceae* (**Figure 1H**) and *Escherichia coli* (*E. coli*) (**Figure 1I**) was also increased with a peak at 24 hours post-irradiation and maintained significantly high at least until 72 hours post-irradiation.

The development of endotoxemia involves gut microbiota dysbiosis and mucosal barrier dysfunction. To determine whether radiation-induced α-defensin down-regulation and microbiota dysbiosis is associated with epithelial barrier dysfunction and endotoxemia, we measured gut permeability *in vivo* and plasma LPS levels. Gut permeability was significantly increased at 4 hours post-irradiation, peaked at 24 hours, and the high permeability sustained at least until 7 days post-irradiation (**Figure 1J**). Similarly, plasma LPS levels were significantly increased by 4 hours post-irradiation, peaked at 24 hours, and remained high until 7 days (**Figure 1K**). These findings indicate that radiation down-regulates intestinal Paneth cell α-defensin expression, paralleled by alteration by microbiota composition, gut barrier dysfunction, and endotoxemia.

### 3.2 Prophylactic HD5 treatment attenuates radiation-induced intestinal dysbiosis

**Figure 2:**
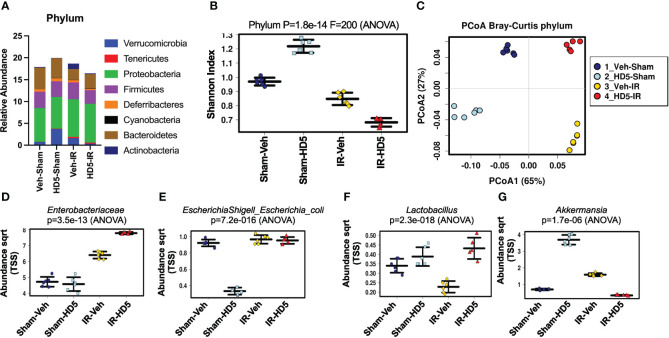
Prophylactic HD5 treatment attenuates radiation-induced intestinal dysbiosis. Adult mice were fed a liquid diet with vehicle (Veh-Sham & Veh-IR) or HD5 (HD5-Sham & HD5-IR) for 24 hours before sham-treatment (Sham) or irradiated (IR). At 24 hours after irradiation, the microbiota composition in colonic flushing was analyzed by 16S rRNA-sequencing and metagenomics. **(A)** The relative abundance of different phyla of bacteria. Data are derived from pooling all values within the group. The experiment was repeated once with similar results. **(B)** The Shannon Index was used to quantify α-diversity. **(C)** Principal coordinate analysis (PCoA) based on Bray-Curtis dissimilarity analysis was performed to determine β-diversity. **(D-G)** Relative abundance of *Enterobacteriaceae* **(D)**, *E. coli* **(E)**, *Lactobacillus* **(F)**, and *Akkermansia* **(G)** in different groups.

HD5, one of the two human Paneth cell α-defensins, was synthesized and tested for antibacterial activity as described before (59). To determine whether α-defensin supplementation in the diet would prevent radiation-induced dysbiosis of gut microbiota, we fed mice a liquid diet with or without HD5 (0.5 mg/ml diet) for 24 hours before irradiation. At 24 hours after irradiation, colonic contents from sham-treated and irradiated mice with or without prophylactic HD5 treatment were analyzed by 16S rRNA sequencing to evaluate the composition of microbial communities (**Figure 2**). Alterations in the relative abundance of multiple bacterial phyla were noted in irradiated animals, including decrease in *Bacteroidetes* abundance and increases in increases in *Verrucomicrobia, Tenericutes*, and *Actinobacteria* (**Figure 2A**). Animals pre-treated with HD5 demonstrated in increase in *Verrucomicrobia* in sham-treated mice, but it showed decreases the abundance of *Verrucomicrobia*, *Firmicutes*, and *Actinobacteria* and an increase in *Bacteroidetes* in irradiated mice (**Figure 2A**). In addition, both irradiated groups (with or without HD5) displayed lower alpha diversity, which is a measure of diversity within each sample, by the Shannon Diversity Index (**Figure 2B**). Bray-Curtis principal coordinate analysis (PCoA) was applied to compare β-diversity, which compares diversity between the sample groups (**Figure 2C**). Both irradiated groups (with or without HD5) were segregated from non-irradiated groups along with principal component 1 (65%, **Figure 2C**), indicating that irradiation profoundly alters microbiota community and prophylactic treatment with HD5 could not prevent this change. However, along with the principal component 2 axis (27%, **Figure 2C**), the microbiota community in mice irradiated with HD5 pre-treatment was most similar to that in non-irradiated sham animals, indicating that HD5 prophylaxis does have a protective effect on the microbiota community structure. Clustering analysis (**Supplementary Figure S1A**) supported the PCoA findings by demonstrating that the non-irradiated animals cluster together on the primary axis (x-axis) but that prophylactic HD5-treated irradiated animals bear significant similarity to untreated controls (y-axis).

The differentially represented taxa within each group were determined by linear discriminant analysis (LDA) effect size (LEfSe), which can identify potential biomarkers and mechanistic targets between groups (**Supplementary Figure S1B**). A*cinetobacter* was the predominant taxa (within the phylum *Psuedomonadota*), distinctly defining the irradiated group. A*kkermansia* was the predominant taxa (within the phylum *Verrucomicrobia*), defining the HD5 prophylaxis group. We analyzed certain specific bacteria taxa based on the above results and known pathogenic and beneficial gut microbiota. Similar to the increase in the abundance of *proteobacteria*, radiation increased *Enterobacteriaceae* (**Figure 2D**) and *E. coli* (**Figure 2E**) regardless of HD5 pre-treatment. Although HD5 reduced *E. coli* in Sham mice, it was not maintained in the face of irradiation. Similar to a decrease in *Firmicutes*, irradiation reduced the abundance of *Lactobacillus* (**Figure 2F**). HD5 prophylactic treatment produced a durable increase in *Lactobacillus* and prevented radiation-induced depletion of *Lactobacillus* (**Figure 2F**). Finally, similarly to *Verrucobacteria*, *Akkermansia* was increased with HD5 prophylaxis. However, this shift did not withstand irradiation-induced change (**Figure 2G**). Of note, analysis of the complete 16S data set revealed that *Akkermansia muciniphila* was the only *Akkermansia* species present in these samples.

Results above analyzed microbiota as relative abundance by 16S rRNA sequencing. In another set of experiments, we evaluated the change in the total amount of specific bacteria in the colonic luminal content by RT-qPCR. Data presented in the supplemental information show that radiation increased *Enterobacteriaceae* (**Supplementary Figure S2A**), *E. coli* (**Supplementary Figure S2B**), and *Akkermansia* (**Supplementary Figure S2C**), while it reduced *Lactobacillus reuteri* (**Supplementary Figure S2D**). HD5 pre-treatment attenuated radiation-induced changes in these specific taxa of bacteria. These data confirmed the changes in microbiota pattern by radiation and HD5 observed by metagenomic analysis. Overall, prophylaxis with HD5 significantly attenuated radiation-induced changes in microbial community structure.

### 3.3 HD5 pre-treatment blocks radiation-induced intestinal epithelial junction disruption, mucosal barrier dysfunction, and inflammatory response

**Figure 3:**
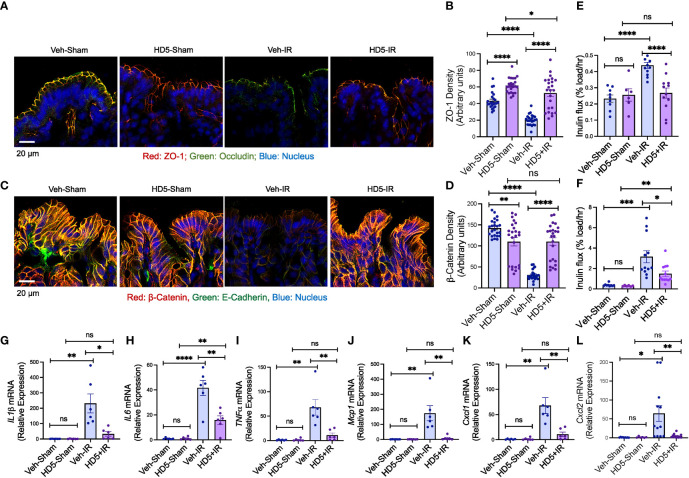
Prophylactic HD5 treatment attenuates radiation-induced colonic mucosal injury. Adult mice were fed a liquid diet with vehicle (Veh-Sham & Veh-IR) or HD5 (HD5-Sham & HD5-IR) for 24 hours before sham-treatment (Sham) or irradiated (IR). TJ and AJ integrity, mucosal permeability, and mucosal inflammatory responses were analyzed 24 hours after irradiation. **(A, B)** TJ integrity was assessed by immunofluorescence staining of colon cryosections for occludin and ZO-1 (green, occludin; red, ZO-1; blue, nucleus) and confocal microscopy. ZO-1 fluorescence density values are presented in panel **(B)**. **(C, D)** AJ integrity was assessed by staining colon sections for E-cadherin and β-catenin (green, E-cadherin; red, β-catenin; blue, nucleus). β-catenin fluorescence density values are presented in panel **(D)**. **(E, F)** Mucosal permeability *in vivo* was evaluated in the colon **(E)** and ileum **(F)** by measuring the vascular-to-luminal flux of FITC-inulin. Values are mean ± sem (n = 6); *p<0.05, **p<0.01, ***p<0.001, and ****p<0.0001 for significant difference between the indicated groups; “ns”, not significant. **(G-L)** At 24 hours after irradiation, total RNA prepared from the colon were analyzed for *IL-1β* **(G)**, *IL-6* **(H)**, *TNFα* **(I)**, *Mcp1* **(J)**, *Cxcl1* **(K)**, and *Cxcl2* **(L)**. Values are mean ± sem (n = 6); *p<0.05, **p<0.01, and ****p<0.0001 for significant difference between the indicated groups; “ns”, not significant.

The epithelial TJ confers intestinal mucosal barrier function. We examined TJ and AJ integrity and evaluated mucosal permeability and cytokine expression to determine the effect of HD5 prophylactic treatment on radiation-induced gut barrier dysfunction and mucosal inflammatory response. Immunofluorescence confocal microscopy showed that radiation reduced occludin and ZO-1 (the TJ proteins) distribution at the epithelial junctions (**Figures 3A, B**), indicating the radiation-induced TJ disruption. HD5 supplementation attenuated the radiation-induced redistribution of occludin and ZO-1, indicating that HD5 prevents radiation-induced TJ disruption. AJ, the protein complex including E-cadherin and β-catenin, is not a physical barrier for macromolecule, but its integrity is essential for maintaining TJ integrity. Radiation caused a loss of junctional distribution of E-cadherin and β-catenin in the colonic epithelium (**Figures 3C, D**), indicating radiation-induced AJ disruption. HD5 supplementation blocked this effect of radiation on AJ. HD5-mediated prevention of TJ and AJ disruption was associated with the significant reduction of radiation-induced mucosal permeability in the colon (**Figure 3E**) and ileum (**Figure 3F**). Radiation increased *IL-1β* (**Figure 3G**), *IL-6* (**Figure 3H**), *TNFα* (**Figure 3I**), *Mcp1* (**Figure 3J**), *Cxcl1* (**Figure 3K**), and *Cxcl2* (**Figure 3L**) mRNA levels in the colonic mucosa. HD5 supplementation significantly dampened radiation-induced increases in cytokine/chemokine expression. These data indicate that HD5 pre-treatment significantly attenuates radiation-induced TJ and AJ disruption, mucosal barrier dysfunction, and the mucosal inflammatory response. HD5 pre-treatment did not prevent radiation-induced body weight loss (**Supplementary Figure S3A**). Histopathologic analyses of the H & E-stained colonic sections showed no significant morphologic changes in the colon (**Supplementary Figure S3B**). In the ileum, radiation showed minor goblet cell loss, cryptitis, mucosal edema, and crypt drop-out regardless of HD5 pre-treatment (**Supplementary Figures S3C, D**).

### 3.4 HD5 therapy 24 hours after irradiation modulates altered gut microbiota composition

**Figure 4:**
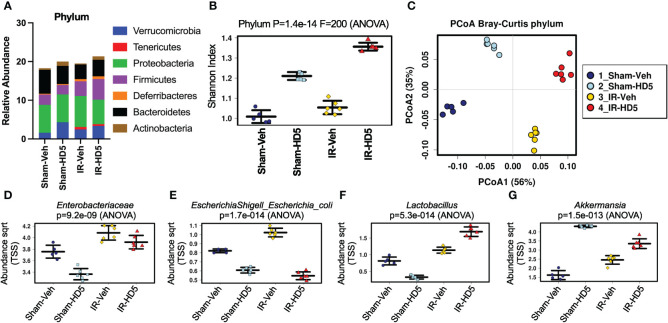
HD5 administered at 24 hours after irradiation modulates altered gut microbiota composition. At 24 hours after sham treatment (Sham) or irradiation (IR), mice were fed a liquid diet with vehicle (Veh-Sham & Veh-IR) or HD5 (HD5-Sham & HD5-IR). After additional 24 hours, the microbiota composition in colonic flushing was analyzed by 16S rRNA-sequencing and metagenomics. **(A)** The relative abundance of different phyla of bacteria. Data are derived from pooling all values within the group. The experiment was repeated once with similar results. **(B)** The Shannon Index was used to quantify α-diversity. **(C)** Principal coordinate analysis (PCoA) based on Bray-Curtis dissimilarity analysis was performed to determine β-diversity. **(D-G)** Relative abundance of *Enterobacteriaceae* **(D)**, *E. coli* **(E)**, *Lactobacillus* **(F)**, and *Akkermansia* **(G)** in different groups.

To evaluate the therapeutic potential of HD5 in radiation-induced alteration of microbial community composition, colonic contents from sham-treated and irradiated mice with or without HD5 therapy (started at 24 hours after irradiation) were analyzed by 16S rRNA sequencing (**Figure 4**). Alterations in the relative abundance of multiple bacterial phyla were noted in the irradiated mice, including decreases in *Bacteroidetes* and *Actinobacteria* and increase*d Firmicutes*, *Verrucomicrobia, Tenericutes, Deferribacteres*, and *Proteobacteria* (**Figure 4A**). HD5 feeding resulted in increases in *Verrucobateria* and *Actinobacteria* and decrease in *Bacteroidetes* in sham-treated mice. Irradiated animals treated with HD5 24 hours post-irradiation demonstrated a decrease in *Proteobacteria*, and an increase in *Verrucomicrobia*, *Firmicutes*, *Deferribacteres*, and *Actinobacteria* (**Figure 4A**). Shannon diversity (alpha-diversity) was unchanged following irradiation alone but increased by HD5 treatment in Sham and irradiated mice (**Figure 4B**). Bray-Curtis principal coordinate analysis (PCoA) of beta-diversity demonstrated that irradiated animals (with or without HD5 treatment) segregated from Sham animals on the principal component 1 (56%, **Figure 4C**). Interestingly, microbiota in HD5-treated samples was more like sham-treated controls than to irradiated mouse samples on the principal component 2 (35%, **Figure 4C**), suggesting that HD5 partially rescues the microbial community changes observed after irradiation. This observation was confirmed by clustering analysis, which demonstrated distinct clusters for irradiated versus non-irradiated animals; HD5-treated mice were more similar in composition to the controls than the irradiated mice (**Supplementary Figure S4A**).

Linear discriminant analysis (LDA) effect size (LEfSe) was used to determine differentially represented taxa within each group (**Supplementary Figure S4B**). At the genus level, a high abundance of *Bacteroides/Parabacteriodes* was the defining feature of the Sham/Control group of mice, whereas a high abundance of Escherichia/Shigella, Pseudomonas, and Enterococcus defined the irradiated group. Furthermore, HD5 treatment in Sham-treated mice was defined by the high abundance of Akkermansia (**Supplementary Figure S4B**), whereas the high abundance of Lactobacillus defined HD5 treatment in irradiated mice. Results from pre-treatment (**Figure 2**) and post-treatment (**Figure 4**) studies confirm that an increased abundance of Verrucomicrobia and Akkermansia is a consistent finding of HD5 supplementation.

Analysis of specific taxa of interest further confirmed that radiation increases the abundance of *Enterobacteriaceae*, which was partially reversed by post-irradiation HD5 treatment (**Figure 4D**). HD5 treatment decreased *E. coli* abundance in both sham-treated and irradiated mice (**Figure 4E**). Irradiation slightly increased *Lactobacillus* abundance (**Figure 4F**). HD5 treatment reduced the abundance of *Lactobacillus* in Sham-treated mice. However, it markedly increased *Lactobacillus* abundance in irradiated mice. Finally, the abundance of *Akkermansia*, driven explicitly by the species *Akkermansia muciniphila*, was increased by irradiation. HD5 treatment increased *Akkermansia* abundance in sham-treated and irradiated mice (**Figure 4G**). Furthermore, RT-qPCR data from an independent experiment showed a reversal of the radiation-induced increase in the amount of *Enterobacteriaceae* (**Supplementary Figure S2A**) and *E. coli* (**Supplementary Figure S2B**) by HD5 post-irradiation treatment. In contrast, HD5 further augmented the radiation-induced increase in *L. reuteri* (**Supplementary Figure S2C**) and *A. muciniphila* (**Supplementary Figure S2D**), supporting the abovementioned metagenomic data.

These findings demonstrate that HD5 treatment can shift the post-irradiated microbial community composition toward the pre-irradiation baseline.

### 3.5 HD5 restores intestinal epithelial integrity and mucosal barrier function following radiation-induced injury

**Figure 5:**
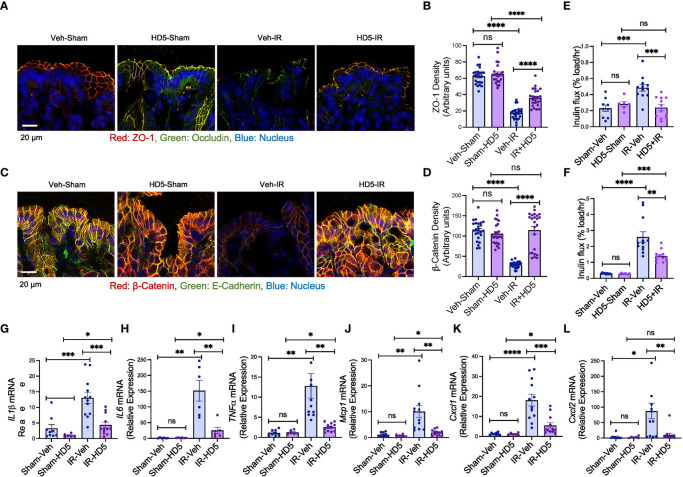
HD5 feeding at 24 hours after irradiation mitigates colonic mucosal injury. At 24 hours after sham treatment (Sham) or irradiation (IR) mice were fed a liquid diet with vehicle (Veh-Sham & Veh-IR) or HD5 (HD5-Sham & HD5-IR). After additional 24 hours, TJ and AJ integrity, mucosal permeability, and mucosal inflammatory responses were analyzed. **(A, B)** TJ integrity was assessed by immunofluorescence staining of colon cryosections for occludin and ZO-1 (green, occludin; red, ZO-1; blue, nucleus) and confocal microscopy. ZO-1 fluorescence density values are presented in panel **(B)**. **(C, D)** AJ integrity was assessed by staining colon sections for E-cadherin and β-catenin. β-catenin fluorescence density values are presented in panel **(D)**. **(E, F)** Mucosal permeability *in vivo* was evaluated in the colon **(E)** and ileum **(F)** by measuring the vascular-to-luminal flux of FITC-inulin. Values are mean ± sem (n = 6); **p<0.01, ***p<0.001, and ****p<0.0001 for significant difference between the indicated groups; “ns” = not significant. **(G-L)** At 48 hours after irradiation (24 hours after start of HD5 treatment), total RNA preparations from the colon were analyzed for *IL-1β* **(G)**, *IL-6* **(H)**, *TNFα* **(I)**, *Mcp1* **(J)**, *Cxcl1* **(K)**, and *Cxcl2* **(L)**. Values are mean ± sem (n = 6); *p<0.05, **p<0.01, ***p<0.001, and ****p<0.0001 for significant difference between the indicated groups; “ns”, not significant.

To determine whether HD5 can reverse the radiation-induced mucosal barrier dysfunction, we examined colonic epithelial TJ and AJ integrity and evaluated colonic mucosal permeability *in vivo* in sham-treated and irradiated mice with or without HD5 administration 24 hours after irradiation. Confocal microscopy showed that HD5 increased the distribution of occludin and ZO-1 at the epithelial junctions in irradiated mice (**Figures 5A, B**), indicating the restoration of TJ integrity. HD5 treatment also increased the junctional distribution of E-cadherin and β-catenin in the colonic epithelium of irradiated mice (**Figure 5C, D**), indicating the restoration of AJ integrity. HD5-mediated restoration of intestinal epithelial TJ and AJ integrity in irradiated mice was associated with a significant reduction of mucosal permeability to inulin in the colon (**Figure 5E**) and ileum (**Figure 5F**). HD5 also attenuated radiation-induced increases in *IL-1β* (**Figure 5G**), *IL-6* (**Figure 5H**), *TNFα* (**Figure 5I**), *Mcp1* (**Figure 5J**), *Cxcl1* (**Figure 5K**), and *Cxcl2* (**Figure 5L**) mRNA levels. These data indicate that HD5, administered 24 hours after irradiation, restores TJ and AJ integrity and mucosal barrier function and attenuates mucosal inflammatory response in irradiated mice. HD5 did not alter the radiation-induced body weight loss (**Supplementary Figure S5A**). There were no significant changes in mucosal morphology in the ileum and colon, irrespective of HD5 treatment (**Supplementary Figure S5B-D**).

To determine the effect of HD5 on small intestinal epithelial TJ integrity, we stained sections of ileum for ZO-1. Prophylactic HD5 treatment significantly blocked radiation-induced redistribution of ZO-1 from the epithelial junctions (**Supplementary Figure S6A**). HD5, administered 24 hours after irradiation, enhanced junctional ZO-1 fluorescence in the ileum of irradiated mice. These results were confirmed by densitometric quantitation of ZO-1 fluorescence (**Supplementary Figure S6B**).

### 3.6 HD5 mitigates radiation-induced endotoxemia and systemic inflammation

**Figure 6:**
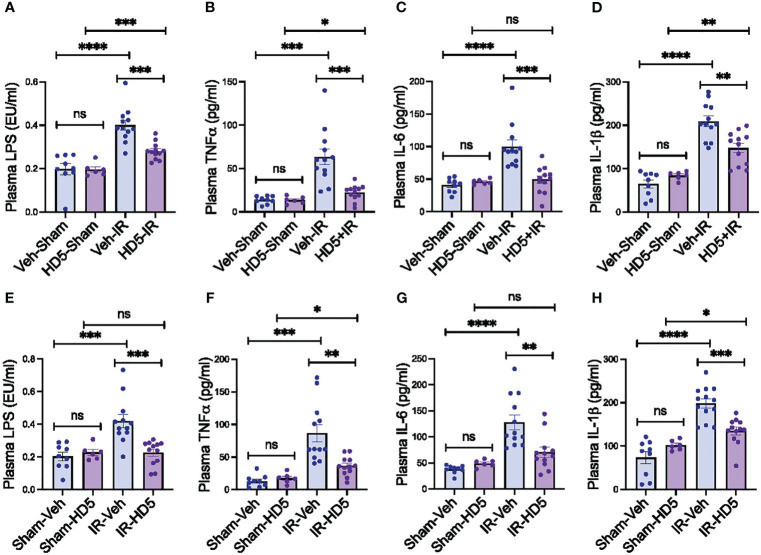
Prevention and reversion of radiation-induced endotoxemia and systemic inflammation by HD5. **(A-D)** Adult mice were fed a liquid diet with vehicle (Veh-Sham & Veh-IR) or HD5 (HD5-Sham & HD5-IR) for 24 hours before sham treatment (Sham) or irradiated (IR). At 24 hours after irradiation, plasma LPS **(A)**, TNFα **(B)**, IL-6 **(C)**, and IL-1β **(D)** were measured. Values are mean ± sem (n = 6); *p<0.05, **p<0.01, ***p<0.001, and ****p<0.0001 for significant difference between the indicated groups; “ns” = not significant. **(E-H)** At 24 hours after sham treatment (Sham) or irradiation (IR), mice were fed a liquid diet with vehicle (Sham-Veh & IR-Veh) or HD5 (Sham-HD5 & IR-HD5). After additional 24 hours, plasma LPS **(E)**, TNFα **(F)**, IL-6 **(G)**, and IL-1β **(H)** were measured. Values are mean ± sem (n = 6); *p<0.05, **p<0.01, ***p<0.001, and ****p<0.0001 for significant difference between the indicated groups; “ns”, not significant.

Dysbiosis of gut microbiota and mucosal barrier dysfunction are two primary factors in developing endotoxemia. In addition, endotoxemia induces systemic inflammation. To determine whether HD5-mediated prevention and reversal of radiation-induced microbiota dysbiosis and gut barrier dysfunction are associated with changes in endotoxemia and systemic inflammation, we measured plasma LPS and cytokine levels. Radiation increased plasma LPS (**Figure 6A**), TNFα (**Figure 6B**), IL-6 (**Figure 6C**), and IL-1β (**Figure 6D**). HD5 pre-treatment significantly attenuated the radiation-induced increase in plasma LPS and cytokine levels (**Figures 6A–D**). HD5, administered 24 hours after irradiation, reduced radiation-induced increase in plasma LPS (**Figure 6E**), TNFα (**Figure 6F**), IL-6 (**Figure 6G**), and IL-1β (**Figure 6H**). These data demonstrate that HD5 supplementation prevents and mitigates endotoxemia and systemic inflammation in irradiated mice.

## 4 Discussion

Gut microbiota dysbiosis and mucosal barrier dysfunction likely lead to endotoxemia in radiation injury. Although the effect of radiation on gut microbiota composition is well recognized, the mechanism by which it alters microbiota composition is unknown. This study presents evidence for the potential role of intestinal Paneth cell dysfunction and α-defensin down-regulation in radiation-induced dysbiosis of gut microbiota and gut barrier dysfunction using a mouse model of radiation injury. First, total body irradiation at 9.5 Gy (LD_50/30_) down-regulates Paneth cell α-defensin expression in the intestine. Second, radiation-induced α-defensin depletion is associated with altered gut microbiota composition, increased intestinal mucosal permeability, and endotoxemia. Third, prophylactic treatment with the human α-defensin HD5 attenuates radiation-induced microbiota dysbiosis and blocks the increase in the abundance of *Verrucomicrobia* and *A. muciniphila*. Fourth, HD5 pre-treatment attenuates radiation-induced TJ disruption, barrier dysfunction, mucosal inflammation, endotoxemia, and systemic inflammation. Finally, therapeutic HD5 administered 24 hours after irradiation significantly reversed radiation-induced microbiota dysbiosis, TJ disruption, barrier dysfunction, endotoxemia, and systemic inflammation.

One of the primary functions of the intestinal Paneth cells is the expression and secretion of α-defensins, which regulate the microbiota composition. This innate immune function of the Paneth cell and α-defensins is crucial in maintaining balanced microbiota composition under physiologic conditions. We show that ionizing radiation down-regulates intestinal α-defensin expression. Radiation reduces α-defensin mRNA in the intestinal mucosa and depletes α-defensin peptides in the colonic lumen. An initial increase in *Defa5* mRNA at four hours post-irradiation suggests an initial upregulation of this innate defense mechanism, which lasted only for a short time. These observations suggest that the downregulation of Paneth cell α-defensins is a potential mechanism involved in radiation-induced dysbiosis of gut microbiota, raising the question of whether α-defensin supplementation prevents or reverses radiation-induced microbiota dysbiosis and tissue injury.

The current study analyzed tissue injury 1 or 2 days after irradiation. At this stage, animals did not show significant morbidity. Their diet intake was unaltered, and their body weights were reduced by about 10%. Therefore, our study addresses the early stage of radiation injury.

Mouse intestinal Paneth cells express at least six isoforms of α-defensins, whereas human Paneth cells produce only two isoforms of α-defensins, HD5 and HD6. HD5 is an antimicrobial peptide that kills pathobionts, likely by forming pores in the bacterial plasma membranes (72). HD5 does not affect the viability of beneficial bacteria such as *L. casei* and *L. plantarum* (59). Therefore, we chose HD5 for this study. Synthetic HD5 was validated for its antimicrobial activity and fed to mice in a well-established liquid diet to test the efficacy of orally delivered HD5 in ameliorating radiation injury. HD5 treatment for 24 hours modified some gut microbiota communities and blocked radiation-induced microbiota dysbiosis. Data suggest that specific microbiota communities are sensitive to HD5; therefore, the microbiota composition in terms of diversity and abundance was modified by HD5 supplementation in irradiated mice. Some taxa of microbiota altered by HD5 in irradiated mice include *Verrucomicrobia*, *Enterobacteriaceae*, *Lactobacillus*, and *Akkermansia*. *Akkermansia* in this study was exclusively represented by *A. muciniphila*. Radiation increased the relative abundance of *A. muciniphila*. Although HD5 by itself elevated the abundance of *A. muciniphila*, it blocked the radiation-induced increase in the abundance of this bacterium. *A. muciniphila* is a well-known second-generation probiotic. The radiation-induced increase in *A. muciniphila* abundance is likely an enhanced defense mechanism in response to altered microbiota or mucosal injury. HD5 may prevent the increase in abundance of *A. muciniphila* by preventing mucosal damage or microbiota alteration.

A previous study indicated that oxidative stress is essential in the radiation-induced disruption of intestinal epithelial TJ and mucosal barrier dysfunction. Alteration of gut microbiota has been shown to affect intestinal epithelial TJ integrity and barrier function (73). Hence, it is likely that radiation-induced microbiota dysbiosis may contribute to TJ and AJ disruption in the colonic epithelium, supported by the current finding that HD5 treatment blocks radiation-induced TJ disruption, mucosal permeability, and inflammatory response. There is no evidence of a direct influence of HD5 on the intestinal epithelium. Therefore, it is likely that the altered gut microbiota causes the HD5-mediated prevention of gut barrier dysfunction and mucosal inflammation. Dysbiosis of gut microbiota and mucosal barrier dysfunction are two main factors that develop endotoxemia, leading to systemic inflammation. Therefore, HD5-mediated prevention of radiation-induced endotoxemia and systemic inflammation is likely caused by the prevention of microbiota dysbiosis and mucosal barrier dysfunction.

This study further investigated the therapeutic potential of HD5 in radiation injury. HD5, when treated 24 hours after irradiation, partially reversed radiation-induced alteration of microbiota composition. HD5 treatment reversed the radiation-induced increase in *E. coli* abundance and decreased in *Lactobacillus* to the extent beyond the basal abundance of these bacteria in the vehicle and sham-treated mice. The abundance of *A. muciniphila* in HD5 pre-treatment and post-treatment studies indicates that increased *A. muciniphila* is a defining biomarker in HD5-treated animals. Although HD5 had no effect or reduced the abundance of *Lactobacillus* in sham-treated mice, *Lactobacillus* is a defining factor in HD5-treated irradiated mice. RT-qPCR analyses indicated *L. reuteri* is a defining biomarker in HD5 treatment in irradiated mice. These data suggest that HD5 treatment may reduce pathogenic bacteria and increase beneficial bacteria in irradiated mice. Reduced expression of Paneth cell α-defensins is a critical mechanism underlying radiation-induced dysbiosis of gut microbiota. Interestingly, a recent study identified α-defensin expression in Goblet cells (74). However, this information needs to be confirmed and validated whether Goblet cell α-defensin is specific to the human intestine or exists in the mouse intestine. Nevertheless, Goblet cell dysfunction in radiation injury is an interesting topic.

HD5, 24 hours after irradiation, restored TJ and AJ integrity and mucosal barrier function. Radiation-induced elevation of mRNA for proinflammatory cytokines and chemokines in the colonic mucosa was reversed by HD5 treatment. The mucosal inflammatory response in the irradiated mice was likely caused by epithelial TJ disruption and translocation of bacterial LPS into the mucosa. Therefore, restoring barrier function by HD5 also leads to the reversal of mucosal inflammation. Consequently, radiation-induced endotoxemia and systemic inflammation were significantly reversed by HD5 treatment. Endotoxemia and systemic inflammation are crucial in multiple organ damage in irradiated animals. The current study suggests that the reversibility of radiation effects on the gut may potentially ameliorate multiple organ injuries.

Immunofluorescence staining of ileum sections for ZO-1 showed that HD5 attenuated radiation-induced epithelial TJ disruption and restored TJ integrity in irradiated mouse ileum. However, minor qualitative differences in the junctional localization of ZO-1 cannot be distinguished by densitometric analysis, as indicated by a partial effect on the ileal mucosal permeability. Our study shows that HD5 generally not suitable to use that adverb effectively reverses radiation injury in the colon but has a partially positive effect in the small intestine. This observation aligns with the hypothesis that HD5 modifies gut microbiota in irradiated mice, restoring epithelial integrity. Since microbiota predominantly resides in the colon, dysbiosis-mediated mucosal injury is a primary mechanism of tissue in irradiated mouse colon, and HD5 heals injury by modifying microbiota composition. Radiation injury in the small intestine may also involve a direct effect of radiation on the highly proliferating crypts cells.

Although the abundance of microbiota in the ileum is about 100,000 folds lower than the microbiota in the colon [reference], ileal microbiota may also play a significant role in regulating ileal mucosal function, and HD5 may have an impact on the ileal microbiota and the epithelial barrier function. Our data from the microbiota composition analyses of colonic flushing do not account for any changes in microbiota composition in the ileum. Therefore, it does not rule out the potential role of altered ileal microbiota composition in the mechanism of barrier dysfunction in irradiated mice. A recent study showed that another antimicrobial peptide, human β-defensin-2, modulated intestinal microbiota composition and attenuated neutrophil infiltration in a graft-versus-host disease model, suggesting a potential direct effect of this peptide on the intestinal mucosa (75). The antibacterial effect of HD5 is attributed to the creation of pores on the bacterial plasma membrane. No direct interaction of HD5 with the intestinal epithelium has been indicated. Therefore, the most likely explanation of HD5-mediated effects on intestinal mucosal functions is an indirect effect of modulating the microbiota composition.

**Figure 7:**
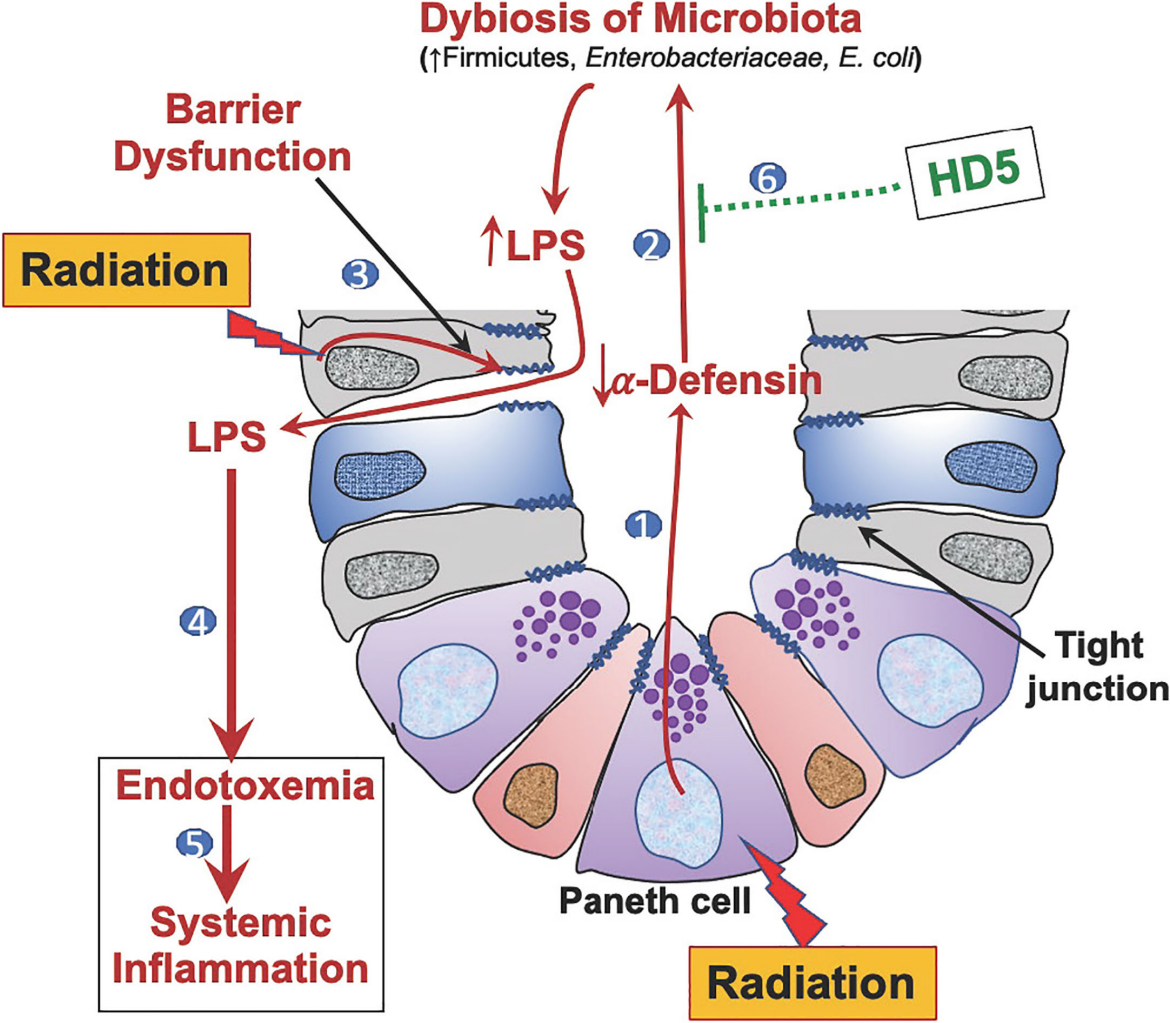
Graphic summary. 1) Radiation-induced Paneth cell dysfunction down-regulates α-defensin expression. 2) α-Defensin depletion leads to dysbiosis of gut microbiota, causing increased LPS production. 3) Radiation disrupts epithelial tight junctions leading to increased LPS translocation. 4) Increased LPS translocation results in endotoxemia. 5) Endotoxemia leads to systemic inflammation. 6) Human defensin-5 (HD-5) supplementation attenuates and mitigates radiation-induced microbiota dysbiosis, endotoxemia, and systemic inflammation.

In summary (**Figure 7**), this study demonstrates that Paneth cell dysfunction and α-defensin depletion are crucial mechanisms underlying radiation-induced gut microbiota dysbiosis, endotoxemia, systemic inflammation, and tissue injury. Human defensin HD5 can prevent and reverse radiation-induced microbiota dysbiosis, gut barrier dysfunction, endotoxemia, and systemic inflammation. Overall, this study identifies the therapeutic potential of HD5 in treating radiation injury.

## Data availability statement

The data presented in the study are deposited in the FigShare repository, DOI: 10.6084/m9.figshare.19758832. Microbiota sequencing data presented in this study are deposited to NCBI BioProject, ID PRJNA839408.

## Ethics statement

The animal study was reviewed and approved by University of Tennessee Health Science Center Institutional Animal Care and Use Committee.

## Author contributions

PS: participated in all experiments and processed data. RGR: started this study and participated in the execution of initial experiments. AM: assisted PS and RR with these experiments and processed data. FG & SB: performed the mass spectrometry and proteomics analyses. SCL: performed irradiation and participated in gut permeability analysis. PR, NS, & CS: performed analyses in small intestinal samples. GT: provided expert advice in designing irradiation model. AG: performed the microbiota analyses, interpreted data, and assisted in manuscript writing. RR: proposed the central hypothesis and designed experimental strategy, PI of funding source, supervising the execution of experiments, data interpretation, and manuscript preparation.

## References

[1] Augustine AD Gondre-Lewis T McBride W Miller L Pellmar TC Rockwell S. Animal models for radiation injury, protection and therapy. Radiat Res (2005) 164(1):100–9. doi: 10.1667/rr3388 15966769

[2] Harari Y Kester D Travis E Wallace J Castro G. Intestinal anaphylaxis: radiation-induced suppression. Am J Physiol (1994) 267(4 Pt 1):G709–15. doi: 10.1152/ajpgi.1994.267.4.G709 7524351

[3] Wang Y Giel-Moloney M Rindi G Leiter AB. Enteroendocrine precursors differentiate independently of wnt and form serotonin expressing adenomas in response to active beta-catenin. Proc Natl Acad Sci U.S.A. (2007) 104(27):11328–33. doi: 10.1073/pnas.0702665104 PMC2040898 17592150

[4] Carbonero F Mayta-Apaza AC Yu JZ Lindeblad M Lyubimov A Neri F. A comparative analysis of gut microbiota disturbances in the gottingen minipig and rhesus macaque models of acute radiation syndrome following bioequivalent radiation exposures. Radiat Environ Biophys (2018) 57(4):419–26. doi: 10.1007/s00411-018-0759-0 30343431

[5] Huang R Xiang J Zhou P. Vitamin D, gut microbiota, and radiation-related resistance: A love-hate triangle. J Exp Clin Cancer Res (2019) 38(1):493. doi: 10.1186/s13046-019-1499-y 31843023 PMC6915920

[6] Jones CB Davis CM Sfanos KS. The potential effects of radiation on the gut-brain axis. Radiat Res (2020) 193(3):209–22. doi: 10.1667/RR15493.1 31898468

[7] Kumagai T Rahman F Smith AM. The microbiome and radiation induced-bowel injury: evidence for potential mechanistic role in disease pathogenesis. Nutrients (2018) 10(10):1405. doi: 10.3390/nu10101405 30279338 PMC6213333

[8] Zhao TS Xie LW Cai S Xu JY Zhou H Tang LF. Dysbiosis of gut microbiota is associated with the progression of radiation-induced intestinal injury and is alleviated by oral compound probiotics in mouse model. Front Cell Infect Microbiol (2021) 11:717636. doi: 10.3389/fcimb.2021.717636 34760714 PMC8573182

[9] Shukla PK Gangwar R Manda B Meena AS Yadav N Szabo E. Rapid disruption of intestinal epithelial tight junction and barrier dysfunction by ionizing radiation in mouse colon in vivo: protection by N-acetyl-L-cysteine. Am J Physiol Gastrointest Liver Physiol (2016) 310(9):G705–15. doi: 10.1152/ajpgi.00314.2015 PMC4867328 26822914

[10] Cohen J. The detection and interpretation of endotoxaemia. Intensive Care Med (2000) 26(Suppl 1):S51–6. doi: 10.1007/s001340051119 10786959

[11] Dey P. Targeting gut barrier dysfunction with phytotherapies: effective strategy against chronic diseases. Pharmacol Res (2020) 161:105135. doi: 10.1016/j.phrs.2020.105135 32814166

[12] Lowry SF. Human endotoxemia: A model for mechanistic insight and therapeutic targeting. Shock (2005) 24(Suppl 1):94–100. doi: 10.1097/01.shk.0000191340.23907.a1 16374380

[13] Schedlowski M Engler H Grigoleit JS. Endotoxin-induced experimental systemic inflammation in humans: A model to disentangle immune-to-brain communication. Brain Behav Immun (2014) 35:1–8. doi: 10.1016/j.bbi.2013.09.015 24491305

[14] van Lier D Geven C Leijte GP Pickkers P. Experimental human endotoxemia as a model of systemic inflammation. Biochimie (2019) 159:99–106. doi: 10.1016/j.biochi.2018.06.014 29936295

[15] Liu J Liu C Yue J. Radiotherapy and the gut microbiome: facts and fiction. Radiat Oncol (2021) 16(1):9. doi: 10.1186/s13014-020-01735-9 33436010 PMC7805150

[16] Tonneau M Elkrief A Pasquier D Paz Del Socorro T Chamaillard M Bahig H. The role of the gut microbiome on radiation therapy efficacy and gastrointestinal complications: A systematic review. Radiother Oncol (2021) 156:1–9. doi: 10.1016/j.radonc.2020.10.033 33137398

[17] Touchefeu Y Franken P Harrington KJ. Radiovirotherapy: principles and prospects in oncology. Curr Pharm Des (2012) 18(22):3313–20. doi: 10.2174/1381612811209023313 22397732

[18] Cui M Xiao H Li Y Zhou L Zhao S Luo D. Faecal microbiota transplantation protects against radiation-induced toxicity. EMBO Mol Med (2017) 9(4):448–61. doi: 10.15252/emmm.201606932 PMC5376756 28242755

[19] Goudarzi M Mak TD Jacobs JP Moon BH Strawn SJ Braun J. An integrated multi-omic approach to assess radiation injury on the host-microbiome axis. Radiat Res (2016) 186(3):219–34. doi: 10.1667/RR14306.1 PMC5304359 27512828

[20] Reis Ferreira M Andreyev HJN Mohammed K Truelove L Gowan SM Li J. Microbiota- and radiotherapy-induced gastrointestinal side-effects (Mars) study: A large pilot study of the microbiome in acute and late-radiation enteropathy. Clin Cancer Res (2019) 25(21):6487–500. doi: 10.1158/1078-0432.CCR-19-0960 31345839

[21] Gerassy-Vainberg S Blatt A Danin-Poleg Y Gershovich K Sabo E Nevelsky A. Radiation induces proinflammatory dysbiosis: transmission of inflammatory susceptibility by host cytokine induction. Gut (2018) 67(1):97–107. doi: 10.1136/gutjnl-2017-313789 28438965

[22] Wang Z Wang Q Wang X Zhu L Chen J Zhang B. Gut microbial dysbiosis is associated with development and progression of radiation enteritis during pelvic radiotherapy. J Cell Mol Med (2019) 23(5):3747–56. doi: 10.1111/jcmm.14289 PMC6484301 30908851

[23] Hollingsworth BA Cassatt DR DiCarlo AL Rios CI Satyamitra MM Winters TA. Acute radiation syndrome and the microbiome: impact and review. Front Pharmacol (2021) 12:643283. doi: 10.3389/fphar.2021.643283 34084131 PMC8167050

[24] Hellweg CE. The nuclear factor kappab pathway: A link to the immune system in the radiation response. Cancer Lett (2015) 368(2):275–89. doi: 10.1016/j.canlet.2015.02.019 25688671

[25] Kusunoki Y Hayashi T. Long-lasting alterations of the immune system by ionizing radiation exposure: implications for disease development among atomic bomb survivors. Int J Radiat Biol (2008) 84(1):1–14. doi: 10.1080/09553000701616106 17852558

[26] Hayashi T Morishita Y Kubo Y Kusunoki Y Hayashi I Kasagi F. Long-term effects of radiation dose on inflammatory markers in atomic bomb survivors. Am J Med (2005) 118(1):83–6. doi: 10.1016/j.amjmed.2004.06.045 15639214

[27] Garg S Boerma M Wang J Fu Q Loose DS Kumar KS. Influence of sublethal total-body irradiation on immune cell populations in the intestinal mucosa. Radiat Res (2010) 173(4):469–78. doi: 10.1667/RR1742.1 PMC2863351 20334519

[28] Santaolalla R Fukata M Abreu MT. Innate immunity in the small intestine. Curr Opin Gastroenterol (2011) 27(2):125–31. doi: 10.1097/MOG.0b013e3283438dea PMC3502877 21248635

[29] Honda K Takeda K. Regulatory mechanisms of immune responses to intestinal bacteria. Mucosal Immunol (2009) 2(3):187–96. doi: 10.1038/mi.2009.8 19262502

[30] Kurashima Y Goto Y Kiyono H. Mucosal innate immune cells regulate both gut homeostasis and intestinal inflammation. Eur J Immunol (2013) 43(12):3108–15. doi: 10.1002/eji.201343782 24414823

[31] Muller CA Autenrieth IB Peschel A. Innate defenses of the intestinal epithelial barrier. Cell Mol Life Sci (2005) 62(12):1297–307. doi: 10.1007/s00018-005-5034-2 PMC11924461 15971105

[32] Wehkamp J Stange EF. Paneth cells and the innate immune response. Curr Opin Gastroenterol (2006) 22(6):644–50. doi: 10.1097/01.mog.0000245541.95408.86 17053443

[33] Beisner J Stange EF Wehkamp J. Paneth cell function–implications in pediatric crohn disease. Gut Microbes (2011) 2(1):47–51. doi: 10.4161/gmic.2.1.14649 21637018

[34] Gassler N. Paneth cells in intestinal physiology and pathophysiology. World J Gastrointest Pathophysiol (2017) 8(4):150–60. doi: 10.4291/wjgp.v8.i4.150 PMC5696613 29184701

[35] Nakamura K Sakuragi N Takakuwa A Ayabe T. Paneth cell alpha-defensins and enteric microbiota in health and disease. Biosci Microbiota Food Health (2016) 35(2):57–67. doi: 10.12938/bmfh.2015-019 27200259 PMC4858879

[36] Salzman NH Underwood MA Bevins CL. Paneth cells, defensins, and the commensal microbiota: A hypothesis on intimate interplay at the intestinal mucosa. Semin Immunol (2007) 19(2):70–83. doi: 10.1016/j.smim.2007.04.002 17485224

[37] Cunliffe RN Mahida YR. Expression and regulation of antimicrobial peptides in the gastrointestinal tract. J Leukoc Biol (2004) 75(1):49–58. doi: 10.1189/jlb.0503249 14525966

[38] Elphick DA Mahida YR. Paneth cells: their role in innate immunity and inflammatory disease. Gut (2005) 54(12):1802–9. doi: 10.1136/gut.2005.068601 PMC1774800 16284290

[39] Rumio C Besusso D Palazzo M Selleri S Sfondrini L Dubini F. Degranulation of paneth cells *via* toll-like receptor 9. Am J Pathol (2004) 165(2):373–81. doi: 10.1016/S0002-9440(10)63304-4 PMC1618569 15277213

[40] Ganz T. Defensins: antimicrobial peptides of innate immunity. Nat Rev Immunol (2003) 3(9):710–20. doi: 10.1038/nri1180 12949495

[41] Ouellette AJ Lualdi JC. A novel mouse gene family coding for cationic, cysteine-rich peptides. Regulation in small intestine and cells of myeloid origin. J Biol Chem (1990) 265(17):9831–7. 2351676

[42] Eisenhauer PB Harwig SS Lehrer RI. Cryptdins: antimicrobial defensins of the murine small intestine. Infect Immun (1992) 60(9):3556–65. doi: 10.1128/iai.60.9.3556-3565.1992 PMC257361 1500163

[43] Ouellette AJ Hsieh MM Nosek MT Cano-Gauci DF Huttner KM Buick RN. Mouse paneth cell defensins: primary structures and antibacterial activities of numerous cryptdin isoforms. Infect Immun (1994) 62(11):5040–7. doi: 10.1128/iai.62.11.5040-5047.1994 PMC303224 7927786

[44] Porter EM Bevins CL Ghosh D Ganz T. The multifaceted paneth cell. Cell Mol Life Sci (2002) 59(1):156–70. doi: 10.1007/s00018-002-8412-z PMC11337504 11846026

[45] Ouellette AJ Darmoul D Tran D Huttner KM Yuan J Selsted ME. Peptide localization and gene structure of cryptdin 4, a differentially expressed mouse paneth cell alpha-defensin. Infect Immun (1999) 67(12):6643–51. doi: 10.1128/IAI.67.12.6643-6651.1999 PMC97078 10569786

[46] Bevins CL. Innate immune functions of alpha-defensins in the small intestine. Dig Dis (2013) 31(3-4):299–304. doi: 10.1159/000354681 24246978

[47] Salzman NH Hung K Haribhai D Chu H Karlsson-Sjoberg J Amir E. Enteric defensins are essential regulators of intestinal microbial ecology. Nat Immunol (2010) 11(1):76–83. doi: 10.1038/ni.1825 19855381 PMC2795796

[48] Sankaran-Walters S Hart R Dills C. Guardians of the gut: enteric defensins. Front Microbiol (2017) 8:647. doi: 10.3389/fmicb.2017.00647 28469609 PMC5395650

[49] Wehkamp J Harder J Weichenthal M Schwab M Schaffeler E Schlee M. Nod2 (Card15) mutations in Crohn's disease are associated with diminished mucosal alpha-defensin expression. Gut (2004) 53(11):1658–64. doi: 10.1136/gut.2003.032805 PMC1774270 15479689

[50] Wehkamp J Koslowski M Wang G Stange EF. Barrier dysfunction due to distinct defensin deficiencies in small intestinal and colonic Crohn's disease. Mucosal Immunol (2008) 1(Suppl 1):S67–74. doi: 10.1038/mi.2008.48 19079235

[51] Camilleri M Madsen K Spiller R Greenwood-Van Meerveld B Verne GN. Intestinal barrier function in health and gastrointestinal disease. Neurogastroenterol Motil (2012) 24(6):503–12. doi: 10.1111/j.1365-2982.2012.01921.x PMC5595063 22583600

[52] Edelblum KL Turner JR. The tight junction in inflammatory disease: communication breakdown. Curr Opin Pharmacol (2009) 9(6):715–20. doi: 10.1016/j.coph.2009.06.022 PMC2788114 19632896

[53] Fukui H. Increased intestinal permeability and decreased barrier function: does it really influence the risk of inflammation? Inflamm Intest Dis (2016) 1(3):135–45. doi: 10.1159/000447252 PMC5988153 29922669

[54] Odenwald MA Turner JR. Intestinal permeability defects: is it time to treat? Clin Gastroenterol Hepatol (2013) 11(9):1075–83. doi: 10.1016/j.cgh.2013.07.001 PMC3758766 23851019

[55] Otani S Coopersmith CM. Gut integrity in critical illness. J Intensive Care (2019) 7:17. doi: 10.1186/s40560-019-0372-6 30923621 PMC6425574

[56] Seo K Seo J Yeun J Choi H Kim YI Chang SY. The role of mucosal barriers in human gut health. Arch Pharm Res (2021) 44(4):325–41. doi: 10.1007/s12272-021-01327-5 33890250

[57] Otani T Furuse M. Tight junction structure and function revisited. Trends Cell Biol (2020) 30(10):805–17. doi: 10.1016/j.tcb.2020.08.004 32891490

[58] Shukla PK Meena AS Gangwar R Szabo E Balogh A Chin Lee S. Lpar2 receptor activation attenuates radiation-induced disruption of apical junctional complexes and mucosal barrier dysfunction in mouse colon. FASEB J (2020) 34(9):11641–57. doi: 10.1096/fj.202000544R PMC7725959 32654268

[59] Shukla PK Meena AS Rao V Rao RG Balazs L Rao R. Human defensin-5 blocks ethanol and colitis-induced dysbiosis, tight junction disruption and inflammation in mouse intestine. Sci Rep (2018) 8(1):16241. doi: 10.1038/s41598-018-34263-4 30389960 PMC6214960

[60] Shukla PK Meena AS Pierre JF Rao R. Central role of intestinal epithelial glucocorticoid receptor in alcohol- and corticosterone-induced gut permeability and systemic response. FASEB J (2022) 36(1):e22061. doi: 10.1096/fj.202101424R 34861075 PMC8647846

[61] Pierre JF Akbilgic O Smallwood H Cao X Fitzpatrick EA Pena S. Discovery and predictive modeling of urine microbiome, metabolite and cytokine biomarkers in hospitalized patients with community acquired pneumonia. Sci Rep (2020) 10(1):13418. doi: 10.1038/s41598-020-70461-9 32770049 PMC7414893

[62] Shukla PK Meena AS Dalal K Canelas C Samak G Pierre JF. Chronic stress and corticosterone exacerbate alcohol-induced tissue injury in the gut-liver-brain axis. Sci Rep (2021) 11(1):826. doi: 10.1038/s41598-020-80637-y 33436875 PMC7804442

[63] Zakrzewski M Proietti C Ellis JJ Hasan S Brion MJ Berger B. Calypso: A user-friendly web-server for mining and visualizing microbiome-environment interactions. Bioinformatics (2017) 33(5):782–3. doi: 10.1093/bioinformatics/btw725 PMC5408814 28025202

[64] Caporaso JG Kuczynski J Stombaugh J Bittinger K Bushman FD Costello EK. Qiime allows analysis of high-throughput community sequencing data. Nat Methods (2010) 7(5):335–6. doi: 10.1038/nmeth.f.303 PMC3156573 20383131

[65] Hughes JB Hellmann JJ Ricketts TH Bohannan BJ. Counting the uncountable: statistical approaches to estimating microbial diversity. Appl Environ Microbiol (2001) 67(10):4399–406. doi: 10.1128/aem.67.10.4399-4406.2001 PMC93182 11571135

[66] Lozupone C Lladser ME Knights D Stombaugh J Knight R. Unifrac: an effective distance metric for microbial community comparison. ISME J (2011) 5(2):169–72. doi: 10.1038/ismej.2010.133 PMC3105689 20827291

[67] Segata N Izard J Waldron L Gevers D Miropolsky L Garrett WS. Metagenomic biomarker discovery and explanation. Genome Biol (2011) 12(6):R60. doi: 10.1186/gb-2011-12-6-r60 21702898 PMC3218848

[68] Basuroy S Sheth P Kuppuswamy D Balasubramanian S Ray RM Rao RK. Expression of kinase-inactive C-src delays oxidative stress-induced disassembly and accelerates calcium-mediated reassembly of tight junctions in the caco-2 cell monolayer. J Biol Chem (2003) 278(14):11916–24. doi: 10.1074/jbc.M211710200 12547828

[69] Parks C Giorgianni F Jones BC Beranova-Giorgianni S Moore BM II Mulligan MK. Comparison and functional genetic analysis of striatal protein expression among diverse inbred mouse strains. Front Mol Neurosci (2019) 12:128(128). doi: 10.3389/fnmol.2019.00128 31178692 PMC6543464

[70] Kansal R Richardson N Neeli I Khawaja S Chamberlain D Ghani M. Sustained B cell depletion by cd19-targeted car T cells is a highly effective treatment for murine lupus. Sci Transl Med (2019) 11(482):eaav1648. doi: 10.1126/scitranslmed.aav1648 30842314 PMC8201923

[71] Mantani Y Nishida M Yuasa H Yamamoto K Takahara E Omotehara T. Ultrastructural and histochemical study on the paneth cells in the rat ascending colon. Anat Rec (Hoboken) (2014) 297(8):1462–71. doi: 10.1002/ar.22937 24788798

[72] Jung SW Lee J Cho AE. Elucidating the bacterial membrane disruption mechanism of human alpha-defensin 5: A theoretical study. J Phys Chem B (2017) 121(4):741–8. doi: 10.1021/acs.jpcb.6b11806 28067516

[73] Roxas JL Viswanathan VK. Modulation of intestinal paracellular transport by bacterial pathogens. Compr Physiol (2018) 8(2):823–42. doi: 10.1002/cphy.c170034 PMC11617015 29687905

[74] Wang Y Song W Wang J Wang T Xiong X Qi Z. Single-cell transcriptome analysis reveals differential nutrient absorption functions in human intestine. J Exp Med (2020) 217(2):e20191130. doi: 10.1084/jem.20191130 31753849 PMC7041720

[75] Ruckert T Andrieux G Boerries M Hanke-Muller K Woessner NM Doetsch S. Human beta-defensin 2 ameliorates acute gvhd by limiting ileal neutrophil infiltration and restraining T cell receptor signaling. Sci Transl Med (2022) 14(676):eabp9675. doi: 10.1126/scitranslmed.abp9675 36542690

