# Supplementary material for: Paneth cell dysfunction in radiation injury and radio-mitigation by human α-defensin 5
Source: Front Immunol. 2023 Aug 10;14:1174140. doi: 10.3389/fimmu.2023.1174140 (PMC10448521; doi:10.3389/fimmu.2023.1174140)
Supplement: Supplementary file 1 [file Presentation_1.pdf]

Supplemental Information

Data Availability Statement
Microbiota metagenomic sequence data are deposited to NCBI BioProject database (NCBI BioProject ID PRJNA839408)
https://submit.ncbi.nlm.nih.gov/subs/bioproject/SUB11490178/overview

Individual values of data presented in different figures in the main article and the supplemental information are deposited to FigShare Data Repository DOI: 10.6084/m9.figshare.19758832

**Table S1:**	**PCR primer sequences**
**Gene**	**5′-3′ Sequence**
23S rDNA (*Enterobacteriaceae*) *En-lsu-1*	Forward: TGCCGTAACTTCGGGAGAAGGCA
	Reverse: TCAAGGACCAGTGTTCAGTGTC
16S rDNA (*E. coli*) *Ecoli*	Forward: CATGCCGCGTGTATGAAGAA
	Reverse: CGGGTAACGTCAATGAGCAAA
L. reuteri	Forward: AGGGTGAAGTCGTAACAAGTAGCC
	Reverse: CCACCTTCCTCCGGTTTGTCA
A. muciniphila	Forward: GAAGACGGAGGACGGAACT
	Reverse: GCGGATTGCTGACGAAGG
Firmicutes	Forward: TGAAACTYAAAGGAATTGACG
	Reverse: ACCATGCACCACCTGTC
Bacteroidetes	Forward: CRAACAGGATTAGATACCCT
	Reverse: GGTAAGGTTCCTCGCGTAT
16S rDNA, (universal)	Forward: ACTCCTACGGGAGGCAGCAGT
	Reverse: ATTACCGCGGCTGCTGGC

Table S2:	PCR primer sequences
**Gene**	**5′-3′ Sequence**
IL-1β	Forward: GCAACTGTTCCTGAACTCAACT
	Reverse: ATCTTTTGGGGTCCGTCAACT
IL-6	Forward: TAGTCCTTCCTACCCCAATTTCC
	Reverse: TTGGTCCTTAGCCACTCCTTC
TNF-α	Forward: CCCTCACACTCAGATCATCTTCT
	Reverse: GCTACGACGTGGGCTACAG
MCP-1/CCL2	Forward: TTAAAAACCTGGATCGGAACCAA
	Reverse: GCATTAGCTTCAGATTTACGGGT
CXCL1	Forward: CTGGGATTCACCTCAAGAACATC
	Reverse: CAGGGTCAAGGCAAGCCTC
CXCL2	Forward: CCAACCACCAGGCTACAGG
	Reverse: GCGTCACACTCAAGCTCTG
Defa5	Forward: GCTCCTGCTCAACAATTCTCC
	Reverse: CAGCTGCAGCAGAATACGAA
Defa6	Forward: GACCAGGCTGTGTCTGTCTC
	Reverse: CCCTTTCTGCAGGTCCCATT
GAPDH	Forward: CTGCACCACCAACTGCTTAG
	Reverse: GGGCCATCCACAGTCTTCT

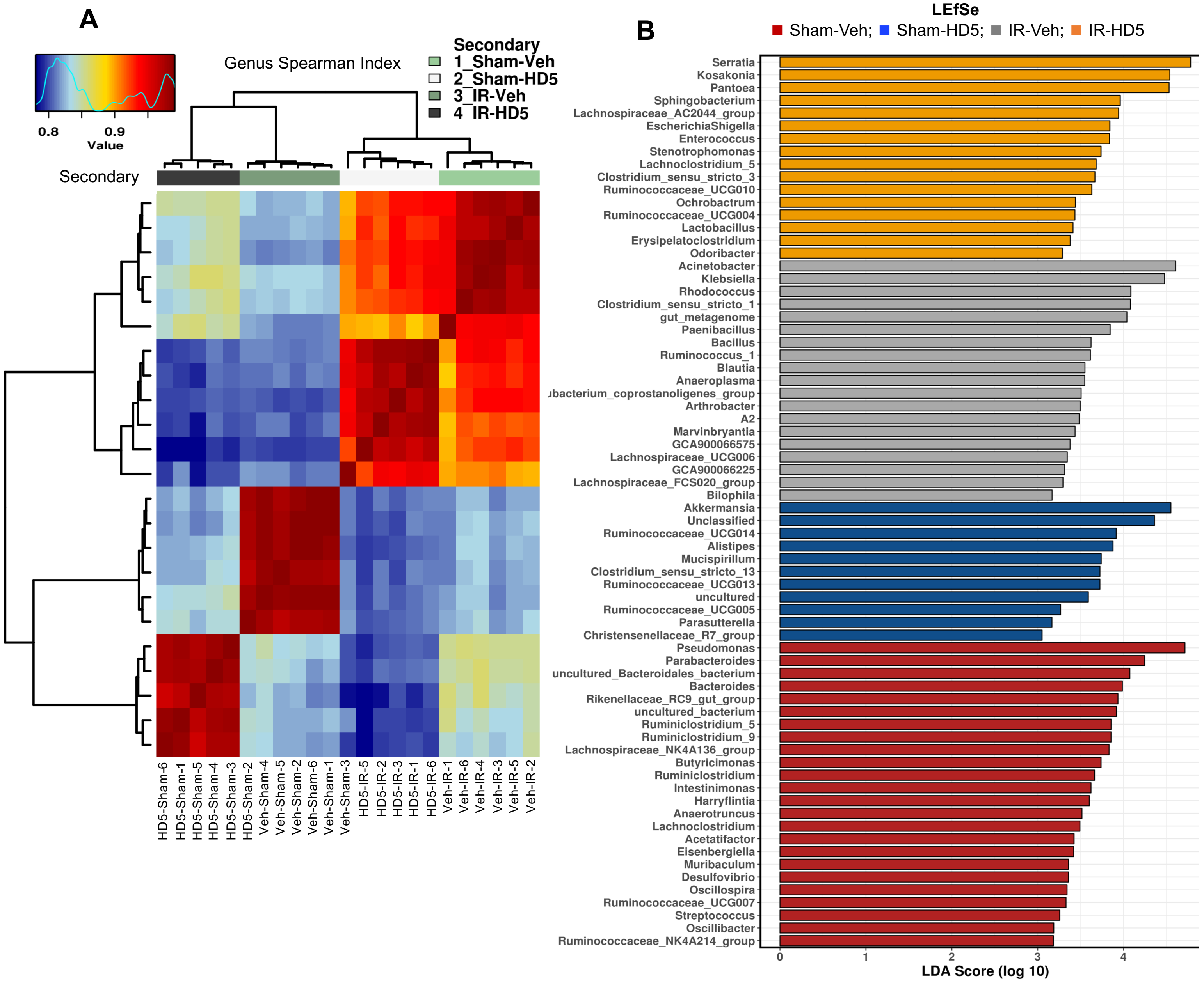

Figure S1: **Prophylactic HD5 treatment attenuates radiation-induced dysbiosis of intestinal microbiota**. Adult mice were fed a liquid diet with vehicle (Veh-Sham & Veh-IR) or HD5 (HD5-Sham & HD5-IR) for 24 hours before sham-treatment (Sham) or irradiated (IR). At 24 hours after irradiation, the microbiota composition in colonic flushing was analyzed by 16S rRNA-sequencing and metagenomics. **A**: Spearman's correlation of microbiota at the genus level in different experimental groups. **B:** Linear discriminate analysis of effect size (LefSe) was used to identify enriched taxa in different groups.

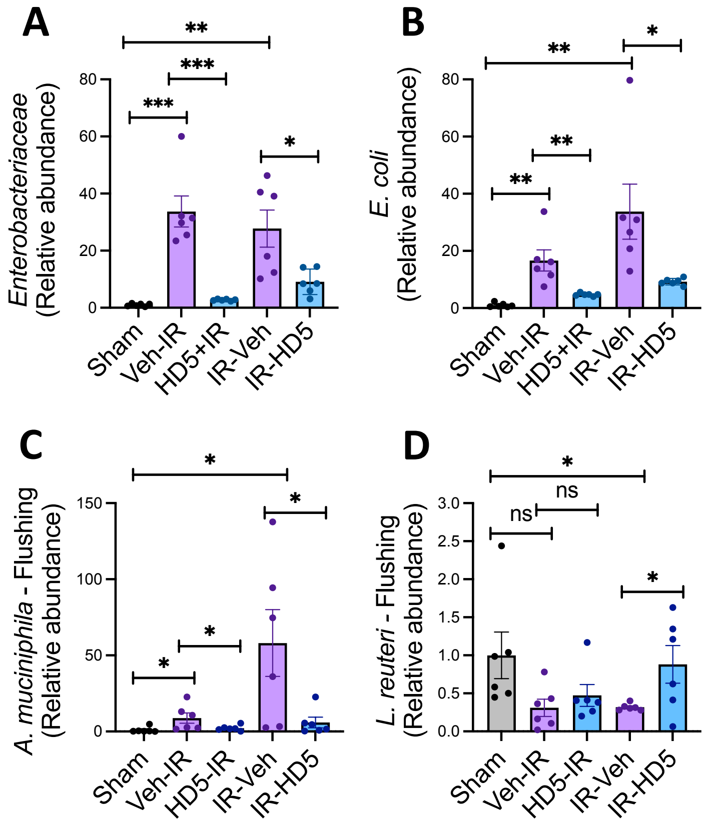

Figure S2: **HD5 prevents and mitigates radiation-induced gut microbiota composition**. Adult mice were subjected to sham treatment (Sham) or irradiation (IR). Some IR groups of mice were fed a liquid diet with vehicle or HD5 (Veh-IR & HD5-IR) for 24 hours before irradiation. Other IR groups of mice were fed a liquid diet with vehicle or HD5 (IR-Veh & IR-HD5) 24 hours after irradiation. DNA preparations from flushing samples were analyzed for the abundance of *Enterobacteriaceae* (A), *E. coli* (B), *A. muciniphila* (C), and *L. reuteri* (D) by RT-qPCR. Values are mean ± sem (n = 6); * = p<0.05, ** = p<0.01, *** = p<0.001 for significant difference between the indicated groups.

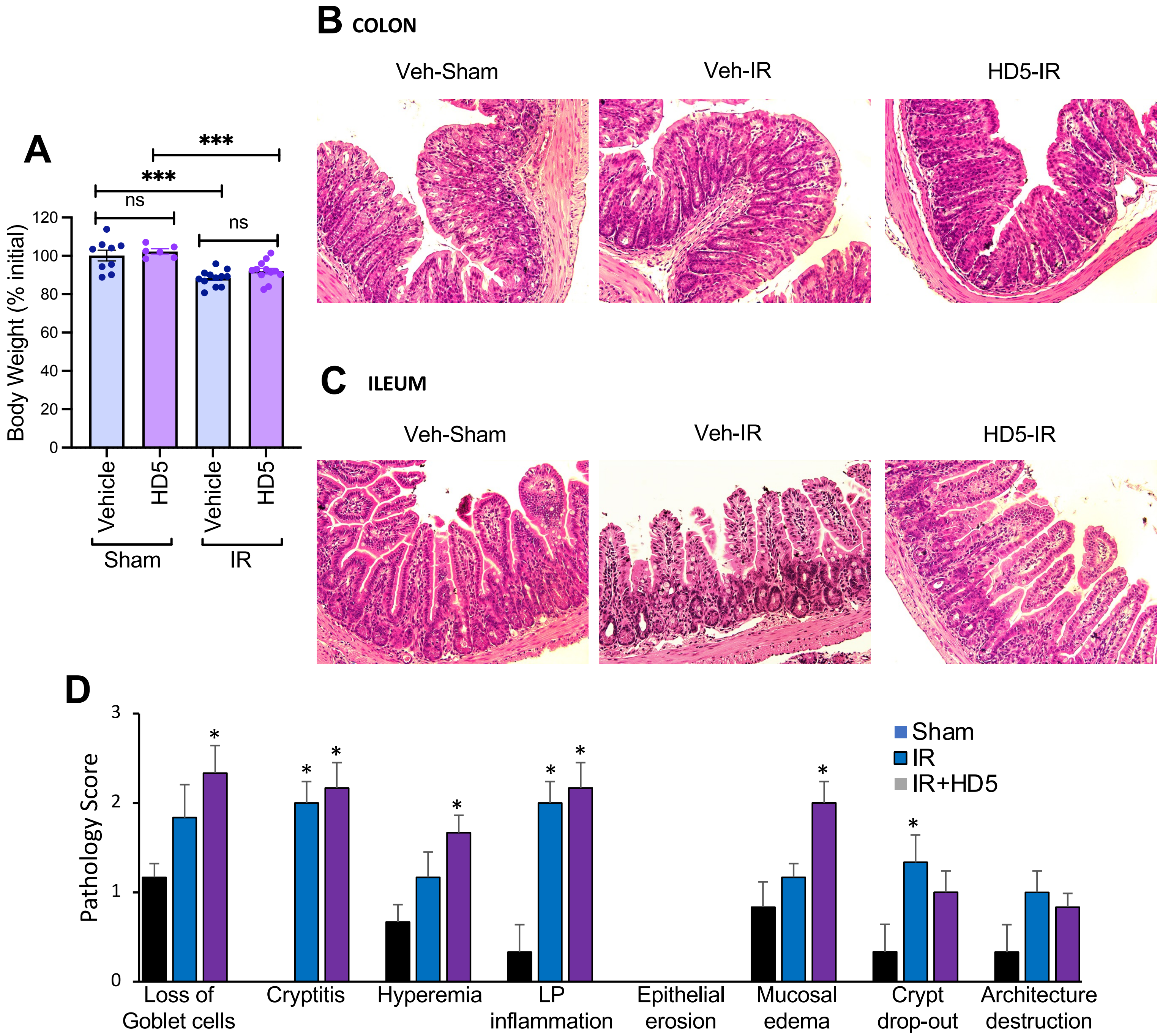

Figure S3: **Effects of radiation and prophylactic HD5 treatment on the intestine.** Adult mice were subjected to sham treatment (Sham) or irradiation (IR). Some IR group of mice were fed a liquid diet with vehicle or HD5 (Veh-IR & HD5-IR) for 24 hours before irradiation. **A.** Body weight changes. **B & C.** Bright field light microscopic images of H & E-stained sections of colon (B) and ileum (C). **D.** Histopathology scores of H & E-stained sections. Values are mean ± sem (n = 6); * = $p<0.05$ for significant difference from corresponding Sham group values.

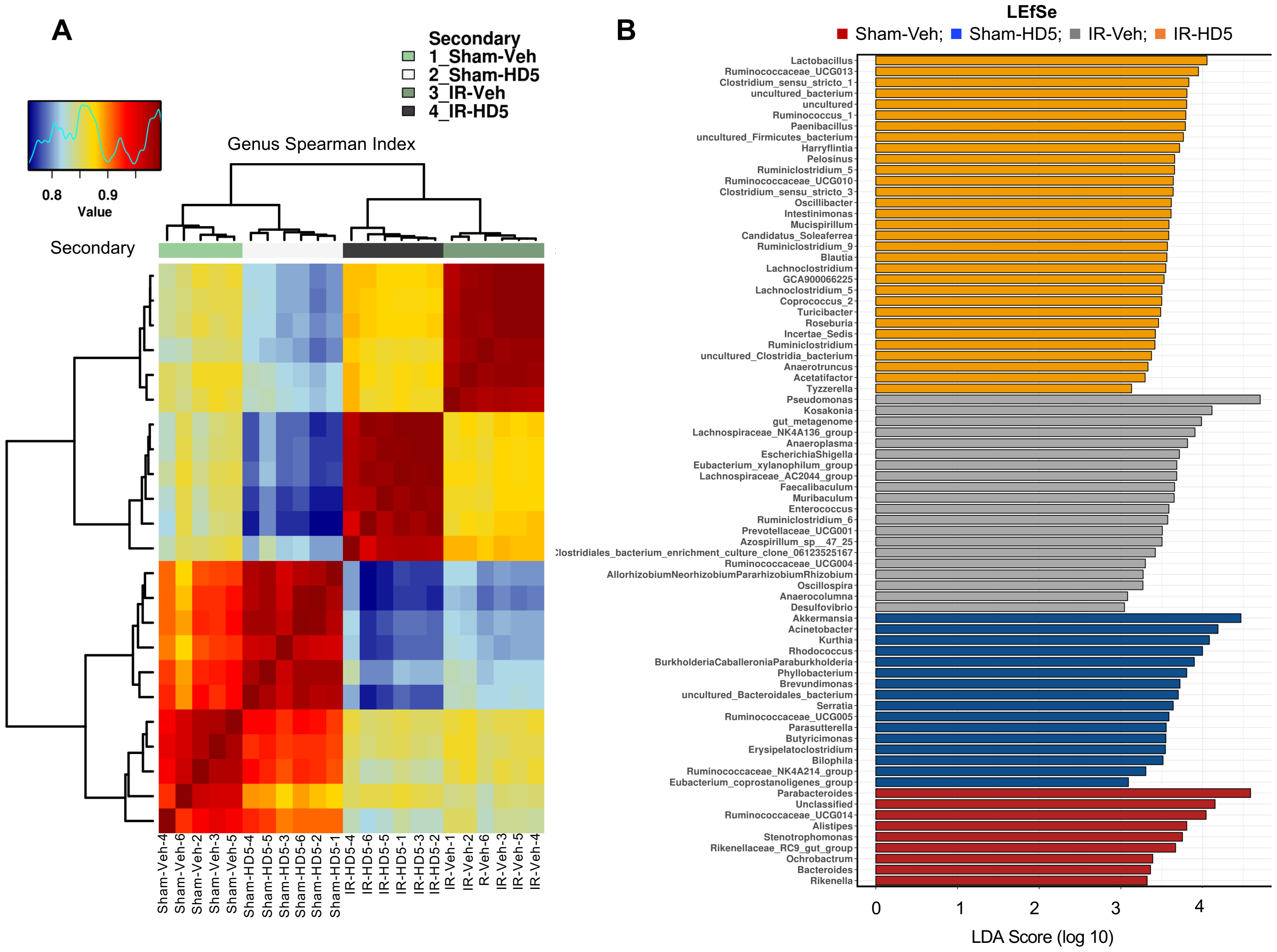

Figure S4: **HD5 at 24 hours after irradiation modulates altered gut microbiota composition**.

At 24 hours after sham-treatment (Sham) or irradiation (IR), mice were fed a liquid diet with vehicle (Sham-Veh & IR-Veh) or HD5 (Sham-HD5 & IR-HD5). After additional 24 hours, the microbiota composition in colonic flushing was analyzed by 16S rRNA-sequencing and metagenomics. **A**: Spearman's correlation of microbiota at the genus level in different experimental groups. **B:** Linear discriminate analysis of effect size (LefSe) was used to identify enriched taxa in different groups.

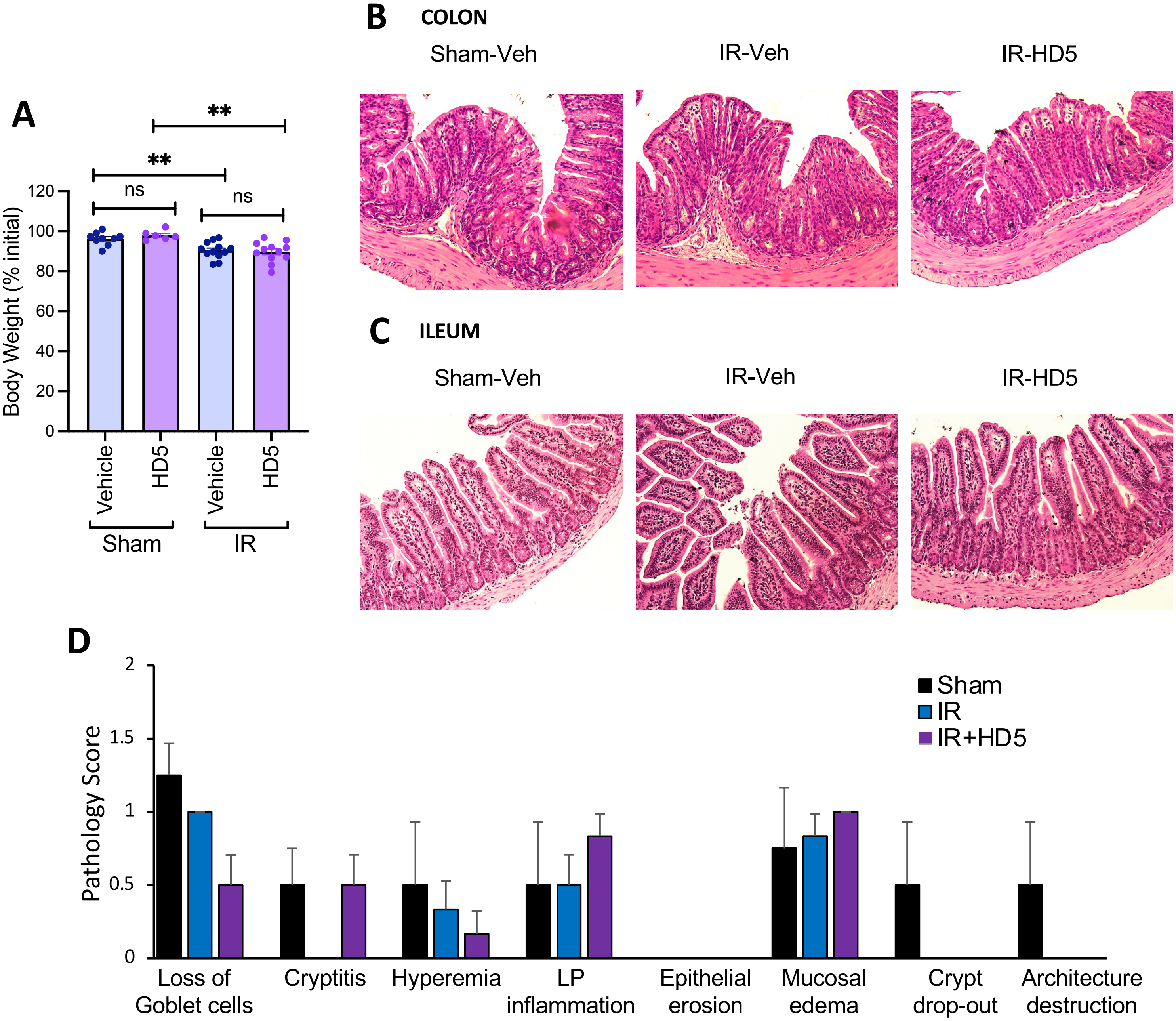

Figure S5: **Effects of radiation and therapeutic HD5 treatment on the intestine.** Adult mice were subjected to sham treatment (Sham) or irradiation (IR). At 24 hours after irradiation, Sham and IR groups of mice were fed a liquid diet with vehicle or HD5 (Sham-Veh & Sham-HD5; IR-Veh & IR-HD5) for 24 hours. **A.** Body weight changes. **B & C.** Bright field light microscopic images of H & E-stained sections of colon (B) and ileum (C). **D.** Histopathology scores of H & E-stained sections. Values are mean ± sem (n = 6).

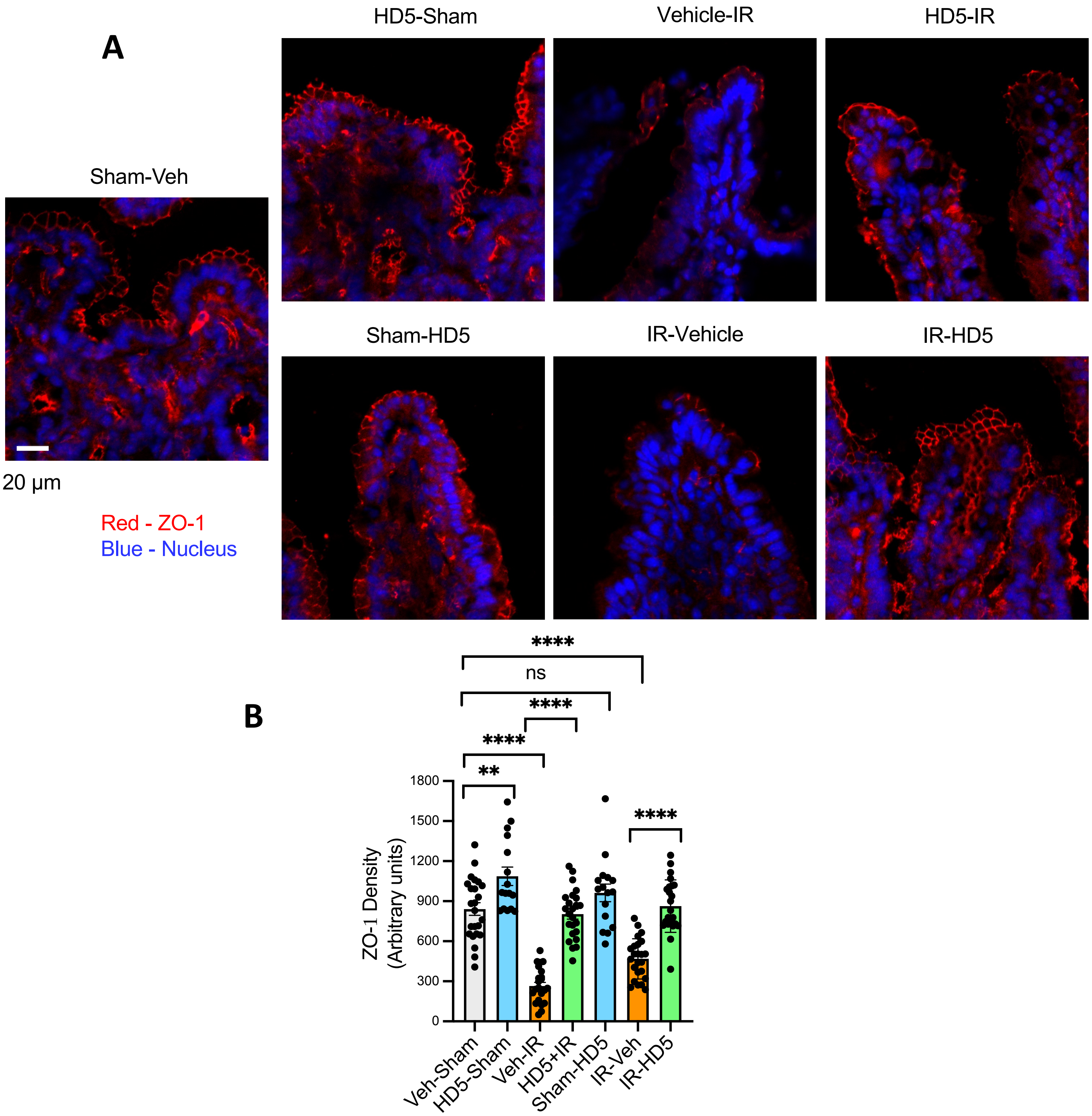

Figure S6: **HD5 treatment reverses radiation-induced epithelial tight junction integrity in the small intestine.**

Adult mice were subjected to sham treatment (Sham) or irradiation (IR). Sham and IR groups of mice were administered Vehicle or HD5 either 24 hours before (HD5-Sham and HD5-IR) or 24 hours after (Sham-HD5 and IR-HD5) irradiation in a liquid diet. At 24 hours after irradiation (HD5-Sham and HD5-IR) or HD5 treatment (Sham-HD5 and IR-HD5) cryosections of ileum were stained for ZO-1 and nucleus. **A.** Representative images of ZO-1 (red) and nucleus (blue). **B.** Densitometric analyses of ZO-1 fluorescence. Values are mean ± sem (n = 8 regions each from 3 mice per group).
